# Polychromic Reporter Mice Reveal Unappreciated Innate Lymphoid Cell Progenitor Heterogeneity and Elusive ILC3 Progenitors in Bone Marrow

**DOI:** 10.1016/j.immuni.2019.05.002

**Published:** 2019-07-16

**Authors:** Jennifer A. Walker, Paula A. Clark, Alastair Crisp, Jillian L. Barlow, Aydan Szeto, Ana C.F. Ferreira, Batika M.J. Rana, Helen E. Jolin, Noe Rodriguez-Rodriguez, Meera Sivasubramaniam, Richard Pannell, James Cruickshank, Maria Daly, Liora Haim-Vilmovsky, Sarah A. Teichmann, Andrew N.J. McKenzie

**Affiliations:** 1 MRC Laboratory of Molecular Biology, Cambridge, CB2 0QH, United Kingdom; 2 Wellcome Trust Sanger Institute, Wellcome Trust Genome Campus, Hinxton, CB10 1SA UK; 3 Theory of Condensed Matter, Cavendish Laboratory, Department of Physics, University of Cambridge, Cambridge, UK; European Molecular Biology Laboratory, European Bioinformatics Institute (EMBL-EBI), Wellcome Genome Campus, Hinxton, UK

**Keywords:** ILC progenitors, ILC development, bone marrow, ILC3 progenitor

## Abstract

Innate lymphoid cells (ILCs) play strategic roles in tissue homeostasis and immunity. ILCs arise from lymphoid progenitors undergoing lineage restriction and the development of specialized ILC subsets. We generated “5x polychromILC” transcription factor reporter mice to delineate ILC precursor states by revealing the multifaceted expression of key ILC-associated transcription factors (Id2, Bcl11b, Gata3, RORγt, and RORα) during ILC development in the bone marrow. This approach allowed previously unattained enrichment of rare progenitor subsets and revealed hitherto unappreciated ILC precursor heterogeneity. *In vivo* and *in vitro* assays identified precursors with potential to generate all ILC subsets and natural killer (NK) cells, and also permitted discrimination of elusive ILC3 bone marrow antecedents. Single-cell gene expression analysis identified a discrete ILC2-committed population and delineated transition states between early progenitors and a highly heterogeneous ILC1, ILC3, and NK precursor cell cluster. This diversity might facilitate greater lineage potential upon progenitor recruitment to peripheral tissues.

## Highlights

• Five-color “polychromILC” transcription factor reporter mice define ILC precursors
• ILC precursors give rise to ILC1, ILC2, and ILC3 and retain NK potential
• A *Rorc*^Kat^ allele allows resolution of extremely rare ILC3 progenitors
• Detection of divergent trajectories for ILC2 and common ILC1, ILC3, and NK development

## Teaser

Innate lymphoid cells (ILCs) play strategic roles in tissue homeostasis and immunity. Walker et al. demonstrate via five-reporter polychrome mice the distinct divergence of ILC2 development from common ILC1, ILC3, and NK progenitors.

## Introduction

Innate lymphoid cells (ILCs) play roles in homeostasis and responses to injury and infection (Colonna, 2018, Eberl et al., 2015, Vivier et al., 2018). ILC subtypes can react rapidly *in situ* to tissue-associated microenvironmental cues, such as those released upon perturbation of mucosal barrier surfaces. The ILC subgroups mirror T helper subsets based on their principal transcription factor (TF) expression and predominant cytokine secretion: ILC1s express T-bet (encoded by *Tbx21*) and produce IFN-γ; ILC2s express high amounts of Gata3 (encoded by *Gata3*) and secrete IL-5 and IL-13; whereas ILC3s express Retinoic-acid-receptor-related orphan nuclear receptor γ (RORγt) (encoded by *Rorc*) and produce IL-17 and IL-22 (Colonna, 2018, Vivier et al., 2018). Furthermore, the previously defined IFN-γ-producing natural killer (NK) cells are closely related to ILC1s but require the TF Eomes for their development, and have profound cytolytic activity (Colonna, 2018, Vivier et al., 2018). ILC1 and NK cells share several surface markers, such as NK1.1 and NKp46. NK subpopulations can also vary within and between tissues, and their classification with regard to ILC1 can prove challenging. It has been suggested that ILC1s, ILC2s, and ILC3s are the innate counterparts of CD4^+^ T cells, whereas NKs are the innate manifestation of cytolytic CD8^+^ T cells.

The similarities with T cells also extend to the plasticity of ILC subsets (Colonna, 2018, Vivier et al., 2018). Thus, depending on tissue microenvironment and inflammatory context, a fraction of ILCs can modulate their cytokine secretion after the differential expression of cytokine-regulating TFs. It has been reported that, in humans, circulating ILC progenitors represent a pool of uncommitted cells with the capacity to acquire the requisite TF and cytokine profiles as they mature in the tissue (Lim et al., 2017). However, defined ILC progenitors can develop in the bone marrow, and circulating naive ILC subsets have also been reported (Krabbendam et al., 2018, Mjösberg and Spits, 2016).

ILC and NK development has been studied extensively in mice (Cherrier et al., 2018, Constantinides et al., 2014, Eberl et al., 2004, Ishizuka et al., 2016, Klose et al., 2014, Wong et al., 2012, Yu et al., 2014, Yu et al., 2016). ILC and NK differentiation requires the expression of inhibitor of DNA binding 2 (Id2) to repress E-box proteins that would otherwise result in T or B cell commitment from common lymphoid progenitors (CLPs). Current models for the differentiation of ILCs from CLPs have suggested a progression of ever more restricted progenitors defined by their cell surface marker and TF expression. However, there is considerable overlap in the phenotypes of these proposed progenitor populations. The earliest ILC progenitors have been defined by the expression of T cell factor 1 (TCF1), TOX, nuclear factor interleukin 3 regulated (NFIL3), alpha4beta7 integrin (α4β7), and Gata3; the early innate lymphoid progenitor (EILP) expresses little or no Id2 or interleukin-7 receptor α (IL-7Rα) (Harly et al., 2018, Yang et al., 2015), and the α-lymphoid precursor (αLP) is Id2^+^ and IL-7Rα^+^ (Yu et al., 2014). EILP and αLP can give rise to NK cells and ILCs. Downstream of these, and reportedly associated with the loss of NK potential, is the common helper-like innate lymphoid progenitor (CHILP) (lineage^−^Id2^hi^IL-7Rα^+^Id2^+^Flt3^−^α4β7^hi^PLZF^+/−^) (Klose et al., 2014) that gives rise to lymphoid tissue inducer (LTi) cells, and the more restricted common ILC progenitor (CILP or ILCP) (lineage^−^Id2^hi^IL-7Rα^+^Flt3^−^α4β7^hi^PLZF^+^) that produces cells committed to one of the “helper” ILC subsets ILC1, ILC2, and ILC3 (Constantinides et al., 2014, Klose et al., 2014). In addition, gene expression analysis of these proposed progenitors indicates that programmed cell death protein-1 (PD-1) is expressed specifically by promyelocytic leukemia zinc finger (PLZF)^+^ ILCP (Seillet et al., 2016), and is a marker of committed ILC progenitors (Yu et al., 2016), but is not essential for ILC development (Seillet et al., 2016). By contrast, several factors have been demonstrated to play roles during the multipotent phases of ILC commitment (Cherrier et al., 2018, Cherrier et al., 2012, Harly et al., 2018, Klose et al., 2014, Lee et al., 2011, Yu et al., 2014, Zhu, 2017), whereas others appear preferentially important for the development of specific ILC subsets, for example the ILC2 progenitor (ILC2P) requires RAR-related orphan receptor α (RORα) (Halim et al., 2012, Wong et al., 2012) and Bcl11b (Califano et al., 2015, Walker et al., 2015, Yu et al., 2015) for its development.

Progress in this area requires the identification, isolation, and characterization of developmental intermediates in order to ascertain their lineage potential and gene expression state. Identification of transitional states is challenging due to the restricted repertoire of cell surface molecules and lack of characteristic markers. Thus, to define intermediate ILC developmental states during lineage segregation in a physiological context, we generated and inter-crossed reporter mouse strains that allow the simultaneous delineation of five key ILC transcription factors, Id2, Bcl11b, Gata3, RORα, and RORγt, in combination with cell surface markers. This approach revealed highly heterogeneous ILC progenitors and allowed for the unprecedented enrichment of specific TF-defined ILCs to facilitate single-cell transcriptomic analysis, leading us to re-evaluate the existing models for ILC differentiation in bone marrow.

## Results

### Generation of Compound Transcription Factor Reporter Mice to Define ILC Lineage Development

To facilitate the identification and isolation of ILC progenitor populations, we generated four reporter mouse strains to trace the expression of the key ILC-associated transcription factors: Id2, Gata3, RORα, and RORγt. Spectrally compatible reporter proteins were selected to enable the generation of compound reporter mice. With the exception of the *Rorc* allele, in which the gene-encoding Katushka (Kat) fluorescent protein was targeted to the translation initiation site for *Rorc* to ensure specificity for the RORγt isoform (Rorc-Kat protein, *Rorc*^Kat^ allele) (Figures S1A and S1B), all other TF reporter alleles comprised a short C-terminal epitope tag, followed by a reporter protein cloned downstream of a T2A self-cleaving peptide. This ensured that the reporter protein was produced stoichiometrically with the endogenous TF: Id2-Blue fluorescent protein (*Id2*^BFP^ allele) (Figures S2A and S2B), Gata3-human CD2 (hCD2) (*Gata3*^hCD2^ allele) (Figures S3A and S3B), and Rora-Teal fluorescent protein (*Rora*^Teal^ allele) (Figures S4A and S4B).

**Figure 1:**
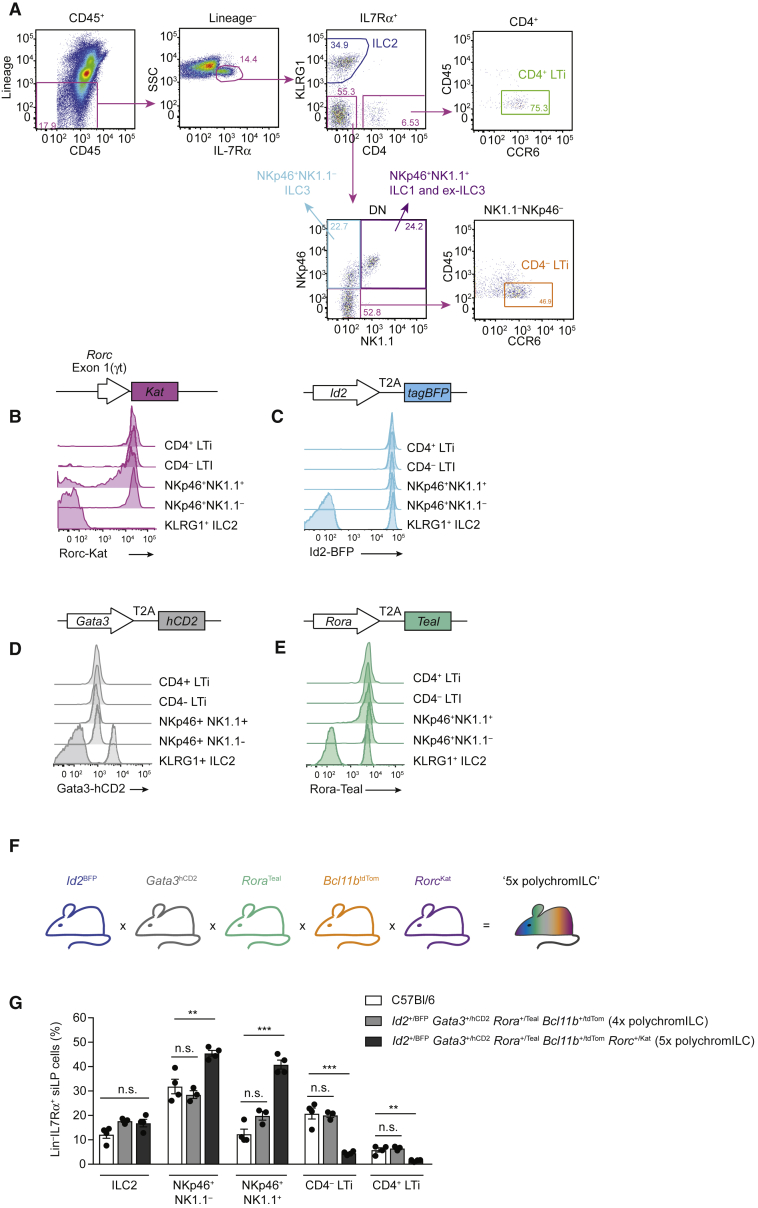
Generation of Compound 5x polychromILC TF Reporter Mice to Define ILC Lineage Development (A) Flow-cytometry gating strategy for ILC subsets in siLP from TF reporter mice (ILC1 or ex-ILC3: CD45^+^Lin^−^IL-7Rα^+^CD4^−^KLRG1^−^NKp46^+^NK1.1^+^; ILC2: CD45^+^Lin^−^IL-7Rα^+^CD4^−^KLRG1^+^; ILC3: CD45^+^Lin^−^IL-7Rα^+^CD4^−^KLRG1^−^NKp46^+^NK1.1^−^; CD4^−^LTi: CD45^lo^Lin^−^IL-7Rα^+^CD4^−^KLRG1^−^NKp46^−^NK1.1^−^CCR6^+^; CD4^+^LTi: CD45^lo^Lin^−^IL-7Rα^+^KLRG1^−^CD4^+^CCR6^+^). (B) Flow-cytometry analysis of Rorc-Kat expression in the ILC subsets of the siLP of *Rorc*^+/Kat^ mice. (C) Flow-cytometry analysis of Id2-BFP expression in the ILC subsets of the siLP of *Id2*^+/BFP^ mice. (D) Flow-cytometry analysis of Gata3-hCD2 expression in the ILC subsets of the siLP of *Gata3*^*+/*hCD2^ mice. (E) Flow-cytometry analysis of Rora-Teal expression in the ILC subsets of the siLP of *Rora*^+/Teal^ mice. (F) Schematic for generation of *Id2*^+/BFP^*Gata3*^*+/*hCD2^*Rora*^+/Teal^*Bcl11b*^+/tdTom^*Rorc*^+/Kat^ 5x polychromILC mice. (G) Flow-cytometric comparison of the ILC subsets in siLP of *Id2*^+/BFP^*Gata3*^*+/*hCD2^*Rora*^+/Teal^*Bcl11b*^+/tdTom^ (4x polychromILC) mice and *Id2*^+/BFP^*Gata3*^*+/*hCD2^*Rora*^+/Teal^*Bcl11b*^+/tdTom^*Rorc*^+/Kat^ (5x polychromILC) mice. Data are representative of 2 independent experiments; mean ± SEM; not significant (ns), ^∗^p < 0.05, ^∗∗^p < 0.01, ^∗∗∗^p < 0.001; one-way ANOVA with Tukey’s post*-*hoc test. Please also see Figures S1–S4.

Rorc-Kat was expressed in LTi cells, NKp46^+^NK1.1^−^ ILC3s and the majority of NKp46^+^NK1.1^+^ ILCs, representing “ILC1 or ex-ILC3” (Figures 1A and 1B). ILC2 did not express Rorc-Kat (Figures 1A and 1B). As described previously for *Rorc*^GFP/GFP^ mice (Eberl et al., 2004), homozygous *Rorc*^Kat/Kat^ mice (which no longer express RORγt) were devoid of peripheral lymph nodes and Peyer’s patches (data not shown) lacked ILC3 and LTi cells (Figure S1C) and exhibited impaired T cell differentiation consistent with *Rorc* expression in double-positive thymocytes (Figures S1D–S1F). *Rorc*^+/Kat^ mice had an increased frequency of NKp46^+^NK1.1^+^ “ILC1 or ex-ILC3” cells in small intestine lamina propria (siLP), consistent with the reported role for RORγt in regulating the balance between ILC3 and ex-ILC3 subsets (Vonarbourg et al., 2010) (Figure S1C). A small Rorc-Kat^−^ cell subset was also present in the NKp46^+^NK1.1^+^ gate, presumably representing ILC1s or ex-ILC3s (Figure S1G). Analysis of the analogous NKp46^+^NK1.1^+^ cell compartment in *Rorc*^Kat/Kat^ mice highlighted the presence of Rorc-Kat^+^ cells, which might represent ILC1s in which the *Rorc* locus is being transcribed, but from which functional RORγt protein cannot be produced (Figure S1G).

Phenotypic analysis of the individual *Id2*^BFP^, *Gata3*^hCD2^, and *Rora*^Teal^ strains revealed minimal perturbation to hematopoietic cell populations (Figures 1A and S2–S4). Consistent with previous reports of *Id2* reporter strains (Jackson et al., 2011, Klose et al., 2014), *Id2*-BFP expression was observed in all mature ILC subsets, LTi cells, and NK cells (Figures 1C and S2C), and gene targeting had no discernible effect on the frequency of the leukocyte populations analyzed (Figures S2D–S2F). Gata3-hCD2 expression was observed in all ILC subsets, being highest in ILC2, and intermediate in all other subsets, consistent with previous reports (Moro et al., 2010, Price et al., 2010, Serafini et al., 2014, Yagi et al., 2014) (Figures 1D and S3C). Analysis of *Gata3*^*+/*hCD2^ and *Gata3*^hCD2/hCD2^ mice indicated little effect on ILC2 frequency or cytokine production after IL-33 challenge (Figures S3D and S3E). All other lymphoid populations examined were unaffected with the exception of a modest increase in the frequency of siLP ILC2s in *Gata3*^hCD2/hCD2^ mice (Figures S3F–S3I).

As anticipated from the critical role for RORα in ILC2 development (Halim et al., 2012, Wong et al., 2012), Rora-Teal was expressed in mature ILC2s and committed bone marrow ILC2Ps (Figures 1E, S4C, and S4D). Although *Rora* gene targeting had no discernible effect on the frequency of mature ILC2s in naive mice, or the expansion and cytokine production of ILC2s upon IL-33 stimulation *in vivo* (Figures S4E–S4G), we noted a reduction in ILC2Ps in *Rora*^Teal/Teal^ mice (Figure S4H), but not in the frequency of CLPs or CHILPs (data not shown). ILC populations in siLP were unperturbed in *Rora*^Teal/Teal^ mice with the exception of ILC3s, which showed a modest skewing toward an NK1.1^+^ “ILC1 or ex-ILC3” phenotype (Figure S4I). All other lymphocyte populations analyzed were normal (Figure S4J).

To analyze ILC progenitors, we inter-crossed the four individual TF reporter mouse strains and additionally incorporated a Bcl11b-tdTomato reporter mouse (Bcl11b-tdTom protein, *Bcl11b*^tdTom^ allele), an important determinant of ILC2 transcriptional programs (Walker et al., 2015, Yu et al., 2015) (Figure 1F). To minimize the influence of altering gene expression through gene targeting, we analyzed compound heterozygotes. Analysis of individual ILC populations from *Id2*^+/BFP^*Gata3*^*+/*hCD2^*Rora*^+/Teal^*Bcl11b*^+/tdTom^ mice indicated that ILC development was unperturbed (Figure 1G). Introduction of the *Rorc*^+/Kat^ allele to give *Id2*^+/BFP^*Gata3*^*+/*hCD2^*Rora*^+/Teal^*Bcl11b*^+/tdTom^*Rorc*^+/Kat^ “5x polychromILC” mice revealed a modest increase in the frequency of the NKp46^+^NK1.1^+^ and NKp46^+^NK1.1^−^ ILC subsets and a reduction in LTi cells (Figure 1G).

Thus, we have generated a panel of individual ILC-associated TF reporter mice that can be intercrossed to produce a combinatorial 5x polychromILC strain for analysis of ILC progenitor development. These mice allow for the specific isolation of viable TF^+^ cell subsets that constitute an extremely minor proportion of bone marrow hematopoietic cells and that can be further analyzed by using *in vitro* assays, *in vivo* adoptive transfers, and single-cell gene expression profiling.

### Rora-Teal Expression Distinguishes between ILCs and NK Cells

**Figure 2:**
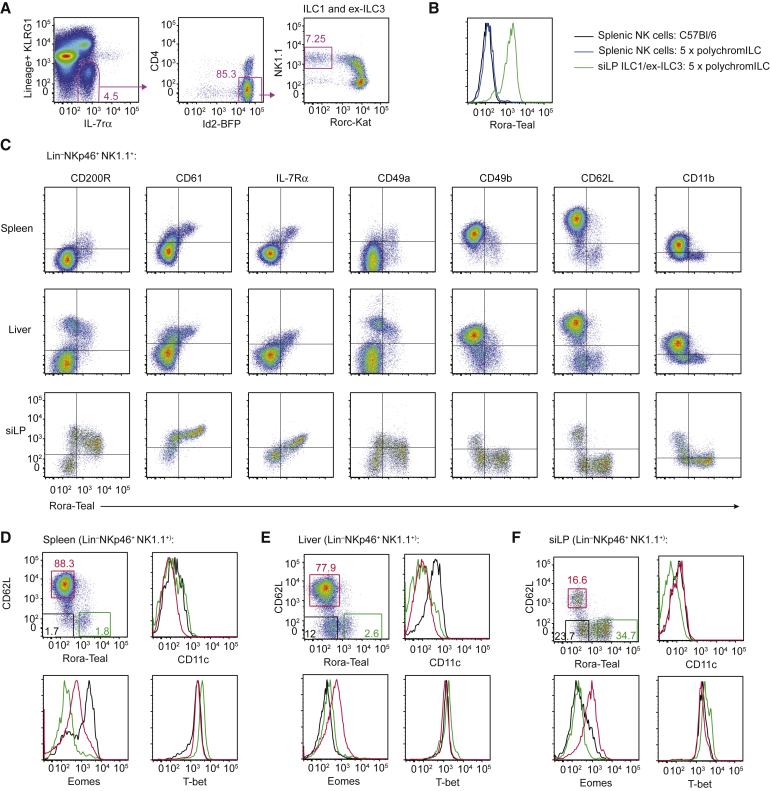
Rora-Teal Expression Distinguishes ILCs from NK Cells (A) Flow-cytometry gating strategy for ILC1 or ex-ILC3 in the siLP of 5x polychromILC mice. (B) Flow-cytometry analysis of Rora-Teal expression in splenic NK cells (CD3^−^CD19^−^NKp46^+^NK1.1^+^) and siLP ILC1or exILC3 (gated as shown in A) from 5x polychromILC mice. (C) Flow-cytometry analysis of the indicated cell surface markers in Lin^−^NKp46^+^NK1.1^+^ cells from the spleen, liver, and siLP of *Rora*^+/Teal^ mice. (D) Flow-cytometry analysis of CD11c, Eomes, and T-bet expression in NK or ILC1 cells from the spleen of *Rora*^+/Teal^ mice. (E) Flow-cytometry analysis of CD11c, Eomes, and T-bet expression in NK or ILC1 cells from the liver of *Rora*^+/Teal^ mice. (F) Flow-cytometry analysis of CD11c, Eomes, and T-bet expression in NK or ILC1 cells from the siLP of *Rora*^+/Teal^ mice. Data are representative of 2 independent experiments.

Rora-Teal in the context of the 5x polychromILC mice revealed that Rora is highly expressed in all ILC populations (data not shown), including siLP Rorc-Kat^−^ ILC1s or ex-ILC3 cells, but not NK cells (Figures 2A and 2B). To establish whether Rora-Teal expression discriminated ILCs from NK cells, we characterized its expression in NK and ILC1s in spleen, liver, and siLP (Figure 2C), as defined previously (Robinette et al., 2015, Weizman et al., 2017). In all tissues, Rora-Teal correlated positively with the ILC1-associated markers CD200R, CD61, IL-7Rα, and CD49a, and negatively with the NK-cell-associated markers CD49b, CD62L, and CD11b (Figure 2C), confirming that *Rora*-Teal was ILC1 restricted. Furthermore RORα-expressing cells were also T-bet^+^ and Eomes^−^, consistent with their classification as ILC1s (Figures 2D–2F and S4K). A third group of IL-7Rα^−^ cells was RORα^−^ and showed a hybrid phenotype that varied considerably between tissues, similar to CD11c^+^ IFNγ-producing NK cells reported previously in the liver after infection (Burt et al., 2008).

### Adoptive Transfer of 5x-polychromILC-Derived Bone Marrow Cells Identifies Heterogeneous Multipotent ILC Progenitors

**Figure 3:**
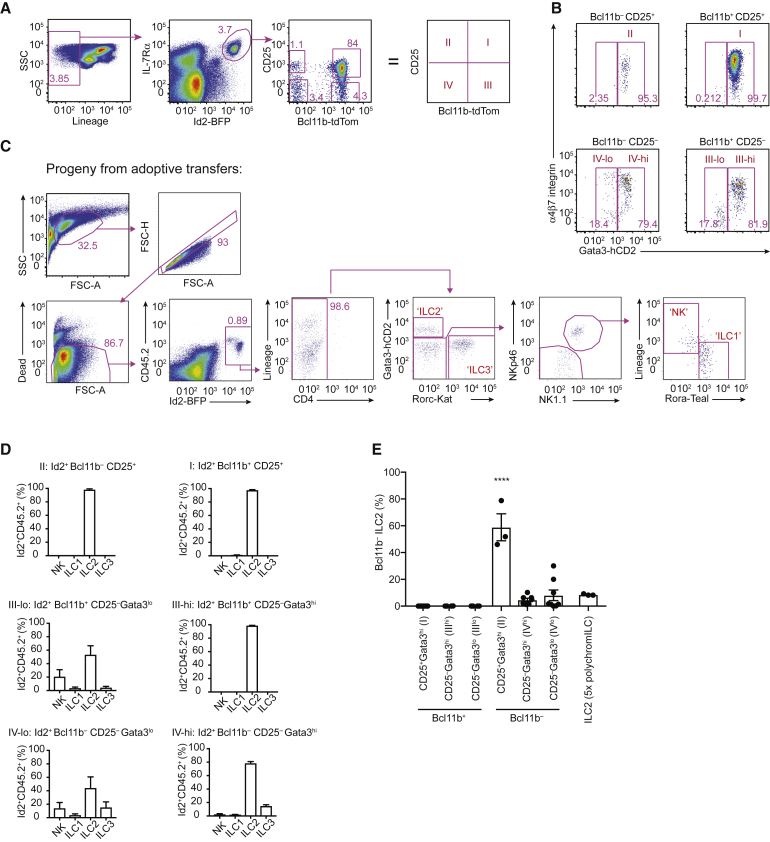
Identification of Heterogeneous Multipotent ILC Progenitors in the Bone Marrow (A) Representative flow-cytometry gating strategy for the characterization and purification of ILC progenitor subsets (populations I, II, III^lo^, III^hi^, IV^lo^, and IV^hi^) (see Table S1) from the bone marrow of 5x polychromILC mice. (B) Flow-cytometry gating strategy for the characterization and purification of ILC progenitor subsets (see A) from the bone marrow of 5x polychromILC mice. (C) Flow-cytometry gating strategy for ILC subsets in siLP arising from adoptive transfer of bone marrow progenitor populations from 5x polychromILC mice into sublethally irradiated *Rag2*^−/−^*Il2rgc*^−/−^ recipients. (D) Flow-cytometry analysis of the ILC progeny generated *in vivo* after the individual adoptive transfers of progenitor cell populations (see A), purified from the bone marrow of 5x polychromILC mice, into *Rag2*^−/−^*Il2rgc*^−/−^ recipients. (E) Proportion of Bcl11b^−^ ILC2 progeny in siLP, derived from the adoptive transfer of purified cell populations (see A) into *Rag2*^−/−^*Il2rgc*^−/−^ recipients. Statistical comparison to ILC2s from a 5x polychromILC mouse. Data are pooled from 2 independent experiments and represent mean ± SEM of 3–11 mice per group; ^∗∗∗∗^p < 0.0001 one-way ANOVA with Dunnett’s post hoc test.

As reported previously, Bcl11b expression in the lineage^−^Id2^+^ subset of bone marrow cells defines a population of ILC2-committed cells (Walker et al., 2015). Using the 5x polychromILC mice, we purified specific progenitor populations on the basis of defined TF reporters and characteristic cell surface receptors. We excluded cells expressing lineage markers (Lin^−^) (see Method Details), then gated for IL-7Rα^+^Id2-BFP^+^ cells, before delineating four populations on the basis of CD25 and Bcl11b-tdTom expression (Figure 3A). The Gata3^hi^ population I (PopI) (Id2^+^Bcl11b^+^CD25^+^) when transferred into sub-lethally irradiated *Rag2*^−/−^*Il2rgc*^−/−^ mice gave rise exclusively to ILC2 progeny (Figures 3B–3D). Thus, ILC2 potential correlated with acquisition of CD25 and Bcl11b^+^. However, there were smaller cell populations within the Lin^−^Id2^+^ subset that expressed only one, or neither, of these markers (Figure 3A). We analyzed their expression of additional TFs, defined by 5x polychromILC mice, and expression of α4β7, which has been reported previously to identify a CHILP (Lin^−^IL-7Rα^+^Id2^+^Flt3^−^α4β7^hi^) from which NK cells were not derived (Klose et al., 2014). PopII (Id2^+^Bcl11b^−^CD25^+^) was also Gata3^hi^ and α4β7^int^ (Figure 3B) and, like PopI, only gave rise to ILC2 progeny upon transfer into *Rag2*^−/−^*Il2rgc*^−/−^ recipients (Figures 3C and 3D), despite their lack of expression of the Bcl11b-tdTom reporter. Although it is possible that these cells were truly Bcl11b^−^, it is also feasible that they expressed only the non-reporter allele of *Bcl11b* as a result of the stochastic nature of Bcl11b expression as reported during T cell development (Ng et al., 2018). Following adoptive transfer, around 50% of the progeny of PopII upregulated td-Tomato expression, suggesting that this “window” for *Bcl11b* allele activation remained open at the CD25^+^ ILC2P stage of ILC differentiation (Figure 3E and data not shown). Notably, subsets that already expressed the Bcl11b reporter allele did not subsequently switch this off, and progeny of *Bcl11b*-tdTom^+^ cells remained tdTomato^+^.

Among Bcl11b-tdTom^+^ cells, there was also a population that was CD25^−^. PopIII (Id2^+^Bcl11b^+^CD25^−^) could be subdivided into a Gata3^hi^α4β7^int^ subset (III^hi^) and a Gata3^lo^α4β7^lo^ subset (III^lo^) (Figures 3A and 3B). When transferred into recipients, PopIII^hi^ was ILC2 restricted (Figure 3D). However, PopIII^lo^ produced mixed progeny including NK cells (defined as NKp46^+^NK1.1^+^RORγt^−^Gata3^lo^RORα^−^) (Figure 3C), ILC2s, and small numbers of ILC1s and ILC3s (Figure 3D). Thus, despite expressing Bcl11b, these cells were not uniformly ILC2-committed and appear to represent a multipotent progenitor, or a mixed population of cells committed to various NK and ILC lineages. PopIV (Id2^+^Bcl11b^−^CD25^−^) was further divided into a Gata3^hi^α4β7^mix^IV^hi^ subpopulation and a Gata3^lo^α4β7^mix^IV^lo^ subset (Figure 3B). The adoptive transfer of PopIV^hi^ generated predominantly ILC2s with a minority of ILC3s, whereas the PopIV^lo^ produced mixed progeny constituting ILC2, ILC3, and NK cells and relatively few ILC1s (Figure 3D).

These results indicate that there are two, or potentially three (i.e., PopII), populations of committed ILC2 progenitors in which the expression of Bcl11b in conjunction with the high expression of Gata3 is associated with ILC2 commitment. When only one of these factors is upregulated, the cells retain multipotency and give rise to NK cells and all ILC populations.

### CHILP-Related Cells Indicate Additional ILC Progenitor Heterogeneity

**Figure 4:**
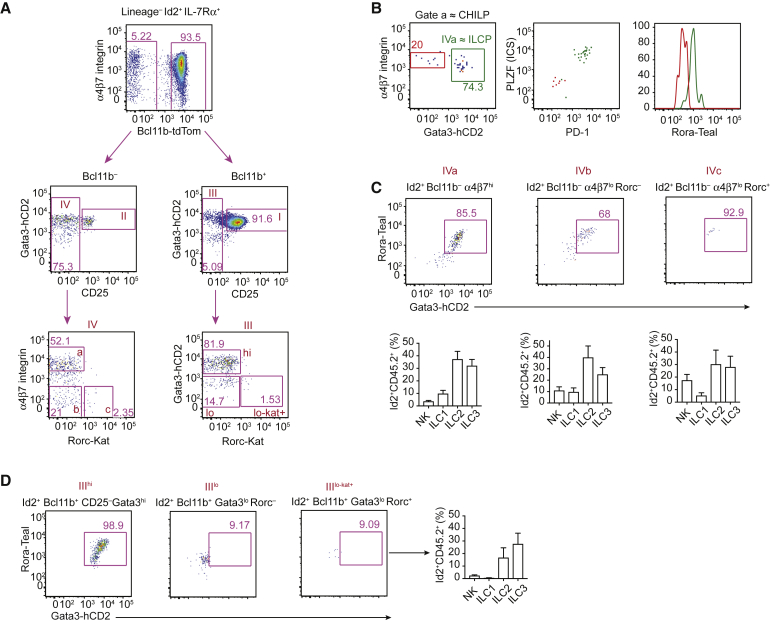
ILCP-Related Cells Indicate Additional ILC Progenitor Heterogeneity (A) Alternative gating strategy for the characterization and purification of populations I, III, and IV from 5x polychromILC bone marrow, to identify Rorc-Kat-positive and -negative progenitors (populations IVa, IVb, IVc, and III^lo-kat+^) (see Table S1). (B) Flow-cytometry analysis of Gata3-hCD2, α4β7, PD-1, PLZF (intracellular stain [ICS]), and Rora-Teal expression within gate IVa (Lin^−^IL-7Rα^+^Id2^+^CD25^−^α4β7^hi^), which corresponds with CHILP (Lin^−^IL-7Rα^+^Id2^+^Flt3^−^α4β7^hi^). The green gate represents Gata3^hi^ subset (Pop IVa); the red gate represents Gata3^lo^ subset. (C) Flow-cytometry analysis of siLP to identify ILC progeny generated *in vivo* after the individual adoptive transfers of progenitor cell populations IVa, IVb, and IVc, purified from the bone marrow of 5x polychromILC mice, into *Rag2*^−/−^*Il2rgc*^−/−^ recipients. (D) Flow-cytometric characterization of progenitor cell subpopulation III^lo-kat+^ and analysis of the ILC progeny in siLP after the adoptive transfer of III^lo-kat+^ progenitors, purified from the bone marrow of 5x polychromILC mice, into *Rag2*^−/−^*Il2rgc*^−/−^ recipients. Data are pooled from 2–4 independent experiments and represent mean ± SEM of 6–14 mice per group.

Having identified multiple populations of ILC progenitors that were not committed to any particular ILC lineage, we examined their transcription factor expression in more detail. We first gated on Lin^−^IL-7Rα^+^Id2^+^ cells then subsetted these on the basis of Bcl11b-tdTom expression with the Bcl11b^−^CD25^−^ cells being equivalent to PopIV and the Bcl11b^+^ cells being divided into PopI and PopIII on the basis of CD25 expression (Figure 4A). Within the Bcl11b^−^CD25^−^ PopIV progenitors, we identified α4β7^hi^ CHILPs and noted that the majority of these cells co-expressed Gata3-hCD2 and Rora-Teal, PD-1, and PLZF, indicating the existence of ILCPs (Figure 4B). Accordingly, these Gata3-hCD2^+^Rora-Teal^+^ ILCPs (also identified herein as PopIVa cells) produced all ILC lineages, but also Rora-Teal^−^ NK cells (Figure 4C). The majority of Bcl11b^−^CD25^−^α4β7^lo^ cells were also Gata3-hCD2^+^Rora-Teal^+^ (PopIVb) and, like IVa, gave rise to mixed progeny of all ILC lineages and NK cells (Figures 4A and 4C). A small fraction (1%–2%) of these Lin^−^IL-7Rα^+^Id2^+^Bcl11b^−^CD25^−^Gata3^hi^α4β7^lo^ cells expressed Rorc-Kat (PopIVc), although this had minimal effect on the progeny obtained from adoptive transfer (Figures 4A and 4C). Rorc-Kat expression within PopIII^lo^ was designated PopIII^lo-kat+^: Lin^−^IL-7Rα^+^Id2^+^Bcl11b^+^CD25^−^Gata3^lo^α4β7^int^RORγt^+^ (Figure 4A). Notably, adoptive transfer of PopIII^lo-kat+^ generated primarily ILC3s and ILC2s and very few ILC1s or NK cells (Figure 4D). We were unable to identify LTi-like cells, as defined by CCR6 expression, from any of the populations analyzed (data not shown).

Together, these results demonstrate that ILCPs, or at least a proportion of ILCPs (or CHILPs), retain the capacity to generate NK cells. Furthermore, 5x polychromILC mice allowed us to identify ILCP-related populations that also generated NK cells and all ILC subsets. Notably, the introduction of the Rorc-Kat reporter enabled the identification of a progenitor with greater restriction to the ILC3 lineage.

### *Zbtb16*-tdTom Reporter Reveals Fluctuating Expression throughout Hematopoiesis

**Figure 5:**
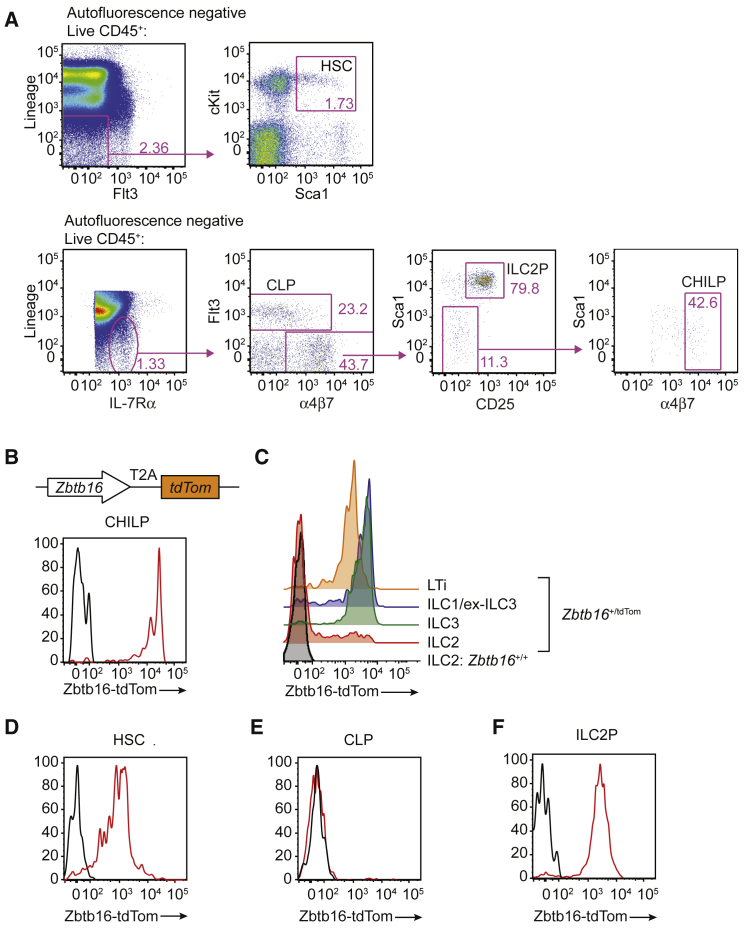
Zbtb16-tdTom Reporter Reveals Fluctuating Expression throughout Hematopoiesis (A) Flow-cytometric gating strategy for HSC, CLP, CHILP, and ILC2P subsets in *Zbtb16*^+/tdTom^ bone marrow. (B) Flow-cytometric analysis of Zbtb16-tdTom in bone-marrow-derived CHILP (Lin^−^CD45^+^IL-7Rα^+^α4β7^hi^Sca1^int/lo^Flt3^−^CD25^−^). (C) Flow-cytometry analysis of Zbtb16-tdTom expression in the ILC subsets in the siLP of *Zbtb16*^*+/*tdTom^ mice. (D) Flow-cytometry analysis of Zbtb16-tdTom expression in bone marrow-derived HSCs (Lin^−^CD45^+^Kit^+^Sca1^+^Flt3^−^). (E) Flow-cytometry analysis of Zbtb16-tdTom expression in bone-marrow-derived CLPs (Lin^−^CD45^+^ IL-7Rα^+^Flt3^+^). (F) Flow-cytometry analysis of Zbtb16-tdTom expression in bone-marrow-derived ILC2Ps (Lin^−^CD45^+^IL-7Rα^+^α4β7^+^Sca1^hi^Flt3^−^CD25^+^). Data are representative of 2 independent experiments. Please also see Figure S5.

It has been reported that PLZF (encoded by *Zbtb16*) expression identifies a subset of CHILPs that are ILC restricted, designated ILCPs, and that LTi cells appear to have no history of PLZF expression as indicated via a *Zbtb16* fate-map and reporter approach, suggesting they originated from a putative PLZF^−^ ILC progenitor (Constantinides et al., 2014). We generated a Zbtb16-tdTom reporter (*Zbtb16*^tdTom^ allele) with the tdTomato protein expressed downstream of a self-splicing T2A sequence (Figures S5A and S5B), in direct relation to PLZF protein (Figure S5C). Analysis of progenitors in the bone marrow and mature ILCs in the siLP indicated no significant effects on the proportions of these cells because of gene targeting (Figures S5D and S5E). Although NKT and NK cells were reduced in *Zbtb16*^tdTom/tdTom^ mice, no detectable changes were observed in immune cell populations measured in the *Zbtb16*^tdTom/+^ mice subsequently used in experiments (Figures S5F and S5G). Zbtb16-tdTom was found to be highly expressed in all CHILPs (Lin^−^IL-7Ra^+^Id2^+^Flt3^−^α4β7^hi^) (Figures 5A and 5B). Furthermore, contrary to a previous report (Constantinides et al., 2014), we found Zbtb16-tdTom in all LTi cells (including fetal LTi), ILC1s, and ILC3s present in the siLP, but not in the majority of mature ILC2s (Figures 5C and S5H). Zbtb16-tdTom reporter expression was also detected in most hematopoietic stem cells (HSCs) (Figures 5A and 5D), but not CLPs (Figures 5A and 5E), indicating fluctuating *Zbtb16* gene expression during hematopoiesis, and expression was also detected in ILC2Ps (Figures 5A and 5F).

### *In vitro* Cell Differentiation Analysis Identifies Multipotent and ILC3-Restricted ILC Progenitors

**Figure 6:**
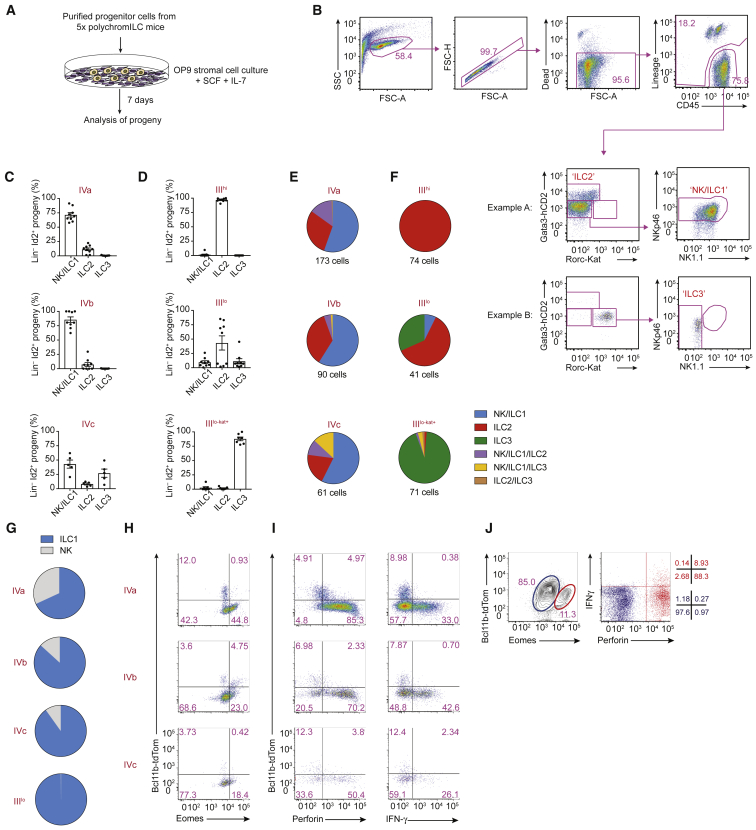
*In Vitro* Analysis Identifies Multipotent and ILC3-Restricted ILC Progenitors (A) Schematic of purified bone marrow progenitor populations co-cultured *in vitro* with OP9 stromal cells to facilitate ILC development. (B) Representative flow-cytometry gating strategy for ILC subsets generated *in vitro* after co-culture of progenitor cell populations, purified from the bone marrow of the 5x polychromILC mice, with OP9 stromal cells. (C) Flow-cytometry analysis of the proportions of ILC subsets generated *in vitro* after co-culture of progenitor cell populations IVa, IVb, and IVc, purified from the bone marrow of 5x polychromILC mice, with OP9 stromal cells. (D) Flow-cytometry analysis of the proportions of ILC subsets generated *in vitro* after co-culture of progenitor cell populations III^hi^, III^lo^, and III^lo-kat+^, purified from the bone marrow of 5x polychromILC mice, with OP9 stromal cells. (E) Characterization of progeny derived from clonal analysis of single IVa, IVb, and IVc progenitor cells, purified from the bone marrow of 5x polychromILC mice, after co-culture with OP9 stromal cells. (F) Characterization of progeny derived from single III^hi^, III^lo^, and III^lo-kat+^ progenitor cells, purified from the bone marrow of 5x polychromILC mice, after co-culture with OP9 stromal cells. (G) Proportion of Eomes^+^ (NK) and Eomes^−^ (ILC1) cells after co-culture of the indicated progenitor populations, purified from the bone marrow of 5x polychromILC mice, with OP9 stromal cells. (H) Flow-cytometric analysis of cells derived from IVa, IVb, and IVc progenitor populations for the expression of Eomes (co-cultured with OP9 stromal cells). (I) Flow-cytometric analysis of cells derived from IVa, IVb, and IVc progenitor populations for the expression of perforin and IFN-γ (co-cultured with OP9 cells and stimulated for 48 hr with IL-2, IL-15, and IL-18). (J) Flow-cytometric analysis of Bcl11b, Eomes, perforin, and IFN-γ expression in LiveCD45.2^+^ spleen cells stimulated *in vitro* with IL-2, IL-15, and IL-18, 6 weeks after transfer of IVa cells into *Rag2*^−/−^*Il2rgc*^−/−^ recipients. (A–D) Data are pooled from 3 independent experiments; mean ± SEM of 5–9 replicate cultures. (E and F) Data are pooled from 3 independent experiments. (G) Data are pooled from 2 independent experiments. (H and I) Data are representative of 3 independent experiments. (J) shows data concatenated from 7 animals taken from 2 independent experiments. Please also see Figure S6.

To complement the *in vivo* adoptive transfer studies, we performed *in vitro* ILC progenitor differentiation assays by using purified progenitor subpopulations from the 5x polychromILC mice. 5x-polychromILC-defined progenitor subsets were co-cultured on OP9 stromal cells with IL-7 and stem cell factor (SCF) to assess their lineage potential (Figure 6A). *In vitro* culture of PopI produced ILC2s (data not shown). However, far greater lineage diversity was observed when assessing the progeny from PopIII and PopIV, similar to results obtained *in vivo*. Cultures of progenitor subsets PopIVa and PopIVb both gave rise predominantly to NK and ILC1 cells and fewer ILC2s and almost no ILC3s (Figures 6B and 6C). PopIVc expressing Rorc-Kat produced almost equivalent proportions of ILC3 and NK or ILC1 cells (Figure 6C). Analysis of PopIII^hi^ and PopIII^lo^ essentially recapitulated the adoptive transfer data, with PopIII^hi^ generating ILC2s and PopIII^lo^ leading to mixed NK and/or ILC1, ILC2, and ILC3 progeny (Figure 6D). PopIII^lo-kat+^ cells were committed almost exclusively to generating Rorc-Kat^+^ ILC3s (Figure 6D).

To address whether the populations giving rise to mixed ILC progeny contained multipotent ILC progenitors or were a mixture of committed cells, we performed single-cell differentiation analysis. The majority of mixed lineage potential cells were located in the Bcl11b^−^ subsets IVa, IVb, and IVc (Figure 6E). A proportion of single cells from PopIVa (and to a lesser extent PopIVb) gave rise to progeny consisting of a mixture of NK and/or ILC1s and ILC2s (Figure 6E). PopIVc also contained multipotent progenitors that gave rise to NK and/or ILC1 and ILC2 cells, but additionally harbored individual progenitors with NK and/or ILC1 and ILC3 potential, consistent with their expression of Rorc-Kat (Figure 6E). By contrast, the Bcl11b^+^PopIII^hi^, PopIII^lo^, and PopIII^lo-kat+^ cells represented lineage-committed precursors (Figure 6F). The Gata3^hi^ cells (PopIII^hi^) gave rise exclusively to ILC2s, whereas the Rorc^+^ (PopIII^lo-kat+^) cells produced almost entirely ILC3s (Figure 6F). The remaining Gata3^lo^Rorc^−^ (PopIII^lo^) cells contained committed progenitors for all three ILC lineages (Figure 6F).

To clarify the proportions of NK cells and ILC1s derived from subsets IVa, IVb, IVc, and III^lo^, we analyzed the expression of Eomes after differentiation in the presence of OP9 stroma. Although all four subsets produced ILC1 progeny (Figure 6G), NK potential, as determined by Eomes expression, was restricted to subsets IVa, IVb, and IVc (Figures 6G and 6H). Furthermore, on stimulation *in vitro* with IL-2, IL-15, and IL-18, the Bcl11b^−^ cells from subsets IVa, IVb, and IVc upregulated perforin and IFN-γ expression, whereas the Bcl11b^+^ cells did not (Figure 6I). The Bcl11b^−^ cells also had the potential to express T-bet (Figure S6A). We also assessed the capacity of progenitor cell populations IVa, IVb, and IVc to give rise to mature NK cells after transfer to *Rag2*^−/−^*Il2rgc*^−/−^ mice. Following engraftment of the donor cells, *ex vivo* stimulation of splenocytes revealed the presence of Eomes, perforin, and IFN-γ^+^ NK cells from PopIVa (Figure 6J), but not IVb or IVc (Figure S6B). Together these data indicate that the IVa progenitor population retains cell-intrinsic NK potential.

Although both the *in vivo* and *in vitro* approaches have their limitations, the *in vitro* data generally support our *in vivo* result but also demonstrate that in a more restricted environment TF^+^ ILC progenitors might show greater lineage restriction. This is notable with the bias toward ILC1 and NK development observed in PopIVa and IVb, and the propensity of Rorc-Kat^+^ populations to generate ILC3s.

### Single-Cell Analysis Identifies the Developmental Divergence of ILC2Ps and Heterogeneous ILC1, ILC3, and NK Progenitors

**Figure 7:**
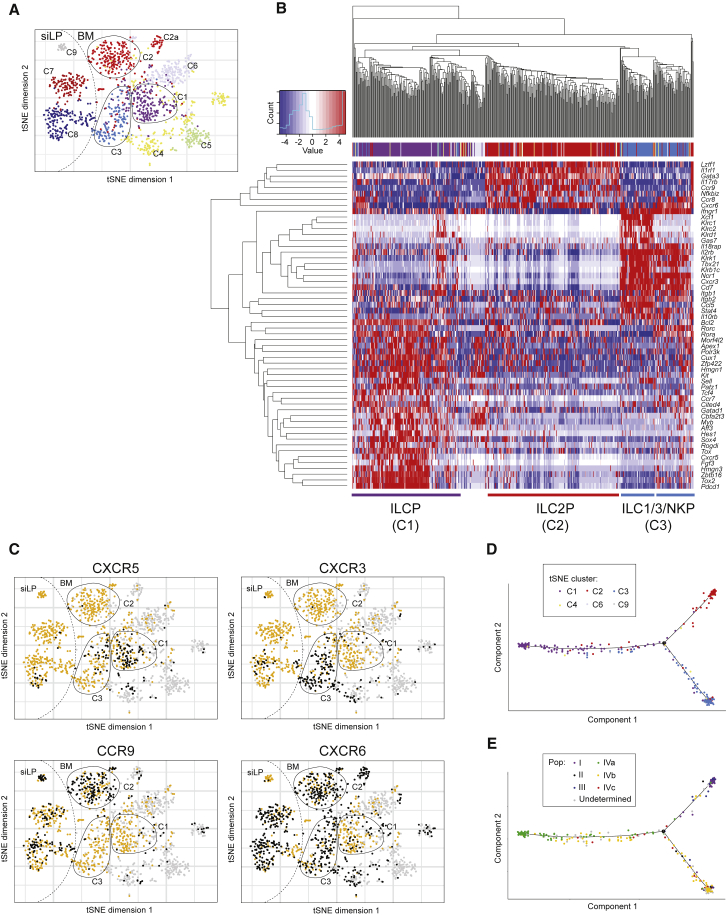
Single-Cell Analysis Identifies Divergence of ILC2P and Heterogeneous ILC3, ILC1, and NK Progenitors (A) tSNE plot of single-cell gene expression analysis from bone marrow and siLP cells (1,637 individual cells) purified from 5x polychromILC mice. Black circles highlight clusters C1–C3. Dotted line divides cells acquired from siLP (left) and BM (right). (B) Heatmap of selected genes differentially expressed between bone marrow cells not expressing *Spi1*, *Gata1*, or *Sox13* (504 cells, predominately cells in clusters C1–C3). (C) tSNE plots of single-cell gene expression of chemokine receptors in the populations defined above in (A) and (B). Black dots indicate cells expressing > 5 normalized pseudocounts for the indicated gene; orange dots indicate cells expressing < 5 normalized pseudocounts for the indicated gene; and light gray dots indicate cells expressing *Spi1*, *Gata1*, or *Sox13*. (D) Cell trajectory from pseudotime analysis of a subset of bone marrow cells not expressing *Spi1*, *Gata1*, or *Sox13* and sequenced with greater read depth (328 cells, predominately cells in clusters C1–C3). Shown is the trajectory from the early progenitor cells located in cluster C1 toward a bifurcation point at which the ILC2P cells (largely cluster C2) and the ILC3, ILC1, and NK cells (largely cluster C3) diverge. (E) Overlay of index-sorted cell phenotype onto the pseudotime trajectory shown in (D). Please also see Figure S7.

To further investigate the heterogeneity of the ILC and NK progenitors, we initially purified individual Lin^−^Id2^+^ bone marrow cells (with a small number of Lin^−^Id2^−^ cells also sampled for comparison) and performed single-cell RNA-sequencing (scRNA-seq) to profile progenitor cell gene expression. Subsequently, to obtain sufficient representation of the extremely rare cells, ILC progenitors were isolated as outlined above and summarized in Table S1. Data were then filtered to include only those cells that expressed the genes encoding *Id2* or *Il7ra*. Clustering identified three predominant cell clusters of ILCs and ILC and/or NK progenitors (clusters C1–C3) as shown on a t-distributed stochastic neighbor embedding (tSNE) plot (Figure 7A), as well as a number of other clusters: C4 and C5 were characterized by the expression of the B cell and/or myeloid gene *Spi1*, whereas C6 expressed the erythroid-specific gene *Gata1*, and a satellite cluster of C2 expressed the γδ T cell-TF *Sox13* (C2a). siLP-derived ILCs were also analyzed to provide comparison to bone marrow samples (clusters C7 and C8). These cells were characterized by the expression of markers more typical of mature ILC2 (cluster C7) or ILC1, ILC3, or NK cells (cluster C8). Analysis of siLP also identified a small cluster of cells expressing immunoglobulin transcripts (C9).

Cluster C1 was characterized by the expression of a spectrum of genes previously associated with early progenitor cell identity, including *Zbtb16*, *Pdcd1*, *Tox*, *Tox2*, *Cbfa2t3*, *Kit*, *Sox4*, *Hes1*, *Gata3*, and *Myb* (Figure 7B). *Cux1* and *Fgf3* were also found to preferentially distinguish cluster C1 (Figure 7B) but have not previously been linked to ILC development. Cluster C2 was characterized by genes associated with the ILC2 lineage, including *Il17rb*, *Il1rl1*, *Lztfl1*, and high expression of *Gata3* (Figure 7B). Cluster C3 was characterized by predominantly ILC1-, ILC3-, and NK-related genes (Figure 7B), indicating a more heterogeneous population and supporting the *in vivo* and *in vitro* progenitor differentiation data. Clustering of expression based on the genes displayed in Figure 7B largely recapitulated the clustering shown in the tSNE plot (Figure 7A), suggesting these genes are among the most important in differentiating these cell types. Notably, we observed additional substructure in the gene expression patterns within cluster C3. One subcluster was characterized by the expression of NK-like genes including *Klr* transcripts, the chemokine *Xcl1*, and the absence of *Rora* and *Rorc*. Whereas the other subcluster contained cells expressing *Rora* and *Rorc* and had an absence of *Klr* genes (Figure 7B). Differential chemokine receptor expression delineated clusters: cells in cluster C1 were predominantly C-X-C motif chemokine receptor 5-positive (*Cxcr5*^+^) but *Cxcr6*^−^, whereas C2 cells expressed principally C-C motif chemokine receptor 9 (*Ccr9*), and *Cxcr3* was largely restricted to C3 (Figure 7C).

To investigate the potential lineage relationship between the progenitors, we performed pseudotime analysis on the bone marrow ILC and NK progenitors by using data from cells that did not express *Spi1*, *Gata1*, or *Sox13*, and that were sequenced with greater read depth (predominately cells in clusters C1–C3). Using this approach, we identified developmental intermediates along a trajectory from the early progenitor cells located in cluster C1 toward a bifurcation point at which the ILC2Ps (cluster C2) and the putative ILC1, ILC3, and NK progenitors (cluster C3) diverged (Figure 7D). Furthermore, using index sequencing data captured during sample collection, we mapped the phenotypes of individual cells onto the pseudotime plot (Figures 7E and S7A). Cluster C2 consisted primarily of cells sorted from population I. The majority of cells in cluster C1 correlated to population IVa, whereas cluster C3 and the transitional states comprised a heterogeneous mix of multiple phenotypes. Altogether these data corroborate our *in vivo* and *in vitro* analyses of NK and ILC developmental potential, demonstrating the existence of Id2^+^ multipotent bone marrow ILC1, ILC3, and NK progenitors. Furthermore, they highlight the heterogeneous potential of the ILC and NK progenitors and how this is distinct from the relatively uniform differentiation of the more frequent ILC2Ps.

## Discussion

Multiple TFs have been associated with ILC differentiation and a number of progenitor states, including the EILP, αLP, CHILP, ILCP, and ILC2P, have been defined on the basis of combinations of surface protein and TF reporter expression (Constantinides et al., 2014, Harly et al., 2018, Klose et al., 2014, Seillet et al., 2016, Walker et al., 2015, Wong et al., 2012, Yang et al., 2015, Yu et al., 2016, Yu et al., 2015). In this study, the generation of 5x polychromILC mice enabled us to delineate bone marrow ILC progenitors on the basis of expression of 5 key ILC-associated TFs: Id2, Bcl11b, Gata3, RORα, and RORγt. This approach enabled the resolution and isolation of rare progenitor cell populations for subsequent analysis of lineage potential and gene expression.

Among the Id2-BFP^+^ bone marrow cells, ILC2Ps were predominant, and Id2-BFP^+^Bcl11b-tdTom^+^Gata3-hCD2^hi^ bone marrow cells gave rise exclusively to ILC2 progeny. Indeed, Bcl11b^+^ and Gata3^hi^ co-expression was sufficient to ensure ILC2 commitment in cells that had not upregulated CD25 (PopIII^hi^). Upregulation of Bcl11b alone was insufficient to drive ILC2 commitment given that Bcl11b^+^Gata3^lo^ cells (III^lo^) retained the capacity to generate all other ILC and NK lineages. A small fraction of this uncommitted Bcl11b^+^Gata3^lo^ cell subset expressed Rorc-Kat (III^lo-kat+^) and had a greater propensity to generate ILC3 upon adoptive transfer and gave exclusively ILC3s upon *in vitro* culture. These elusive cells (approximately 1 cell in a million in the bone marrow) appear to represent the earliest committed ILC3 progenitors in mouse bone marrow, and their discovery demonstrates the utility of our multi-TF-driven approach. Although ILC progenitors might seed the peripheral tissues and differentiate *in situ* to populate the ILC3 niche, our results now raise the possibility that already-committed bone-marrow-derived ILC3 progenitors populate mucosal tissues. Future studies will be needed to determine the relative contributions of these alternative pathways for ILC3 generation.

We also identified a spectrum of multipotent Id2^+^Bcl11b^−^ bone marrow populations that were heterogeneous with respect to α4β7 integrin and Gata3 expression. A major fraction of these cells was α4β7^hi^Gata3^+^ (IVa) and co-expressed PLZF and PD-1 and corresponded to the ILCP reported previously to be an ILC1-, ILC2-, ILC3-restricted progenitor (Constantinides et al., 2014, Harly et al., 2018). However, we observed that in addition to generating ILC1s, ILC2s, and ILC3s, these ILCPs gave rise to a population of Rora-Teal^−^lineage^+^NK1.1^+^NKp46^+^ NK cells that expressed Eomes and perforin, indicating additional lineage potential. Thus ILCP, as currently defined, is not exclusively ILC-lineage restricted but retains some NK lineage potential. A recent report utilizing Id2 and PLZF reporters has also demonstrated that an ILCP population has the potential to differentiate into ILC1, ILC2, ILC3, and NK cells (Xu et al., 2019). We also identified a minor fraction of α4β7^lo^Gata3^+^ ILCP-related cells (IVb), a small proportion of which also expressed Rorc-Kat (IVc) that also gave rise to a population of *Rora*-Teal^−^Lin^+^NK1.1^+^NKp46^+^ NK cells, in addition to ILC1s, ILC2s, and ILC3s. Although a small proportion of α4β7^lo^ ILCP-related cells expressed Rorc-Kat, unlike their Bcl11b^+^ counterparts, these cells showed little propensity for ILC3 restriction after stromal cell co-culture or adoptive transfer. Rather than signifying commitment, Rorc-Kat expression in this instance could reflect the multi-lineage priming reported to occur prior to ILC-lineage commitment during embryogenesis (Ishizuka et al., 2016).

The expression of PLZF has been used to define the transition between CHILP and ILCP (Constantinides et al., 2014, Klose et al., 2014) and has been associated with an ILC-committed progenitor population that has lost the capacity to generate NK cells and LTi. It has been postulated that LTi cells originate from PLZF^−^ progenitors on the basis of cell fate analyses (Xu et al., 2019) and the observation that mature LTi are negative for both fate-mapping and expression reporters in a strain that drives expression of a Cre-GFP fusion protein from the *Zbtb16* promoter as part of a bicistronic allele utilizing an IRES sequence (Constantinides et al., 2014). However, our data indicate that the vast majority of HSCs, CHILPs, and mature LTi cells are Zbtb16-tdTom^+^. Similarly, EILPs have also been reported to be PLZF^+^ (Harly et al., 2018). It is thus surprising that LTi cells were fate-mapper negative in the PLZF-fm strain (Constantinides et al., 2014). This discrepancy might reflect a lower expression of the Cre-GFP fusion protein and/or inefficiency of Cre excision of the STOP cassette, which triggers fate-mapping reporter expression, or might be attributable to the requirement to remove FM^+^ bone marrow (BM) cells and reconstitute recipients with FM^−^ cells because of PLZF expression during mouse development (Constantinides et al., 2014). By contrast, our *Zbtb16*^tdTom^ strain showed a wide dynamic range of reporter expression (CHILPs > ILC2Ps > HSCs > CLPs), which accurately recapitulated the profile of PLZF protein expression determined by intracellular staining. Incorporation of the *Zbtb16*^tdTom^ allele into additional compound reporter strains might help to clarify the role of PLZF in ILC commitment.

Single-cell gene expression analysis of ILC bone marrow progenitors revealed three predominant clusters of cells with distinct ILC-associated gene expression patterns: an early progenitor (C1), an ILC2P population (C2), and a mixed ILC1, ILC3, and NK progenitor population (C3). C1 was characterized by genes associated with early progenitors including *Zbtb16*, *Pdcd1*, *Tox*, *Tox2*, *Kit*, *Sox4*, *Hes1*, *Gata3*, and *Myb* (Allan et al., 2017, Cherrier et al., 2018, Eberl et al., 2004, Ishizuka et al., 2016, Lee et al., 2011, Seehus et al., 2015, Wong et al., 2012, Yu et al., 2016, Zhu, 2017). Indeed, the widespread expression of *Zbtb16* (PLZF) and *Pdcd-1* (PD-1) in C1 suggests they are ILCPs (population IVa in our analysis). PD-1 has been reported as a marker of PLZF^+^ ILCPs (Seillet et al., 2016, Yu et al., 2016), though the absence of PLZF (Harly et al., 2018) or PD-1 in mice did not alter ILCP development (Seillet et al., 2016). C1 was also characterized by the expression of transcriptional repressors such as core binding factor Cbfa2t3 (Seillet et al., 2016) and *Cux1*, a repressor for which there are multiple binding sites in the *Zbtb16* promoter region (Fréchette et al., 2010) but which has not been linked previously with ILC progenitors. Cluster 2 was clearly ILC2Ps, characterized by the expression of the *Il17rb* and *Il1rl1* encoding the receptors for IL-25 and IL-33 and high amounts of *Gata3* (Halim et al., 2012, Yu et al., 2016, Zhu, 2017). *Lztfl1*, the gene encoding leucine zipper transcription-factor-like 1 (LZTFL1), is reported to be expressed in all mature ILC subsets in the small intestine but here was largely restricted to the ILC2Ps. Cluster 3 represented a highly heterogeneous population which shared both ILC1, ILC3, and NK cell gene expression signatures. Two subclusters were present in C3. The putative ILC1 and ILC3 progenitors frequently co-expressed *Tbx21* and *Rorc*, in addition to *Ncr1* (encoding NKp46), suggesting their potential to generate either lineage. The other subcluster was characterized by the expression of *Klr* genes and *Tbx21*, but not *Rorc or Rora*, highlighting their probable commitment to the NK cell lineage. C3 was most clearly delineated from ILCPs and ILC2Ps by the expression of CD7, a cell surface molecule used as a pan-ILC marker in human studies (Lim et al., 2017) that is poorly studied in mouse ILC biology. Although a small number of genes were expressed by most cells in C3, this cluster was otherwise notable for the heterogeneity of gene expression.

Clusters C1, C2, and C3 could be discriminated by their chemokine receptors. As shown previously, CXCR6 expression defines a proportion of EILPs and αLPs (Yang et al., 2015, Yu et al., 2014); however, CXCR6^−^ αLPs also appear capable of generating ILCs and NK cells (Yu et al., 2014). Our single-cell analysis indicated that only relatively few cells in cluster C1 expressed *Cxcr6* but that almost all cells in clusters C2 and C3 were *Cxcr*6^+^, indicating that this marker has limited specificity among ILC resident bone marrow progenitors. However, it is notable that the deletion of CXCR6 results in a modest accumulation of ILCPs in the bone marrow (Chea et al., 2015). A recent study reported that *Cxcr5* expression, assessed by bulk cell population gene expression analysis, was absent in αLP, moderately induced in EILP, and highest in ILCPs and surface CXCR5 was detected on ILCPs (Harly et al., 2018). With the benefit of 5x polychromILC mice to enrich, identify, and isolate rare single progenitors, we identified that *Cxcr5* expression was mostly restricted to C1 and there was little to no expression detected in C2 or C3. This suggests that Cxcr5 expression is a useful marker of the ILCPs. However, it is unclear what role CXCR5 is playing in ILC development. CXCR3 has been reported to be expressed on mature ILC1s (Cortez et al., 2015, Klose et al., 2014), although its role in ILC progenitor biology is unknown. As reported previously, CCR9 is primarily expressed on ILC2Ps (Hoyler et al., 2012), as we observed in cluster C2 in the bone marrow, and ILC2 homing to the small intestine is impaired in *Ccr9*^−/−^ mice (Kim et al., 2015).

These clusters, defined by gene expression, mirror the bone marrow progenitors identified during the phenotypic characterization of 5x polychromILC mice. Our pseudotime analysis suggests that the switch associated with ILC2 lineage commitment represents a rapid and concerted alteration in gene expression leading to “all-or-nothing” lineage commitment with very few cells representing intermediates along the ILC2P branch. In contrast, many more transition states were located between the early progenitor (ILCP) and putative ILC1, ILC3, and NK progenitors, reflecting the heterogeneity in TF and surface molecule expression that we observed among non-ILC2P bone marrow progenitors. A recent report has described a similar divergence of human ILC2 progenitors from a progenitor with shared NK cell and ILC3 lineage potential (Chen et al., 2018).

Our data support a model whereby EILPs progress directly to the PLZF^+^ ILCP state, then progress through a trans-ILCP intermediate phase before expressing TFs characteristic of lineage-committed progenitors and divergence of ILC2Ps from a common NK, ILC1, and ILC3 developmental pathway (Figure S7B).

## STAR★Methods

### Key Resources Table

**Table:** 

REAGENT or RESOURCE	SOURCE	IDENTIFIER
**Antibodies**		

Anti-mouse CD3 (145-2C11)	Biolegend	Cat#100320; RRID: AB_312685
Anti-mouse CD5 (53-7.3)	eBioscience	Cat#25-0051-81; RRID: AB_657755
Anti-mouse CD4 (GK1.5)	eBioscience	Cat#25-0041-82; RRID: AB_469576
Anti-mouse CD8 (53-6.7)	eBioscience	Cat#25-0081-82; RRID: AB_469584
Anti-mouse CD11b (M1/70)	Biolegend	Cat#101216; RRID: AB_312799
Anti-mouse CD11c (N418)	eBioscience	Cat#25-0114-82; RRID: AB_469590
Anti-mouse CD19 (eBio1D3)	eBioscience	Cat#25-0193-82; RRID: AB_657663
Anti-mouse FcεRI (MAR-1)	eBioscience	Cat#25-5898-82; RRID: AB_2573493
Anti-mouse Ly-6C/Ly-6G (RB6-8C5)	eBioscience	Cat#25-5931-82; RRID: AB_469663
Anti-mouse TER-119 (TER-119)	eBioscience	Cat#25-5921-82; RRID: AB_469661
Anti-mouse NK1.1 (PK136)	eBioscience	Cat#25-5941-82; RRID: AB_469665
Anti-mouse Nkp46 (29A1.4)	Biolegend	Cat#137619; RRID: AB_2562452
Anti-mouse IFN-γ (XMG1.2)	Biolegend	Cat#505838; RRID: AB_2629667
Anti-mouse CD16/CD32 (Fc Block) (2.4G2)	BioXCell	Cat#BE0307; RRID: AB_2736987
Anti-mouse IL-7Rα (SB/199)	Biolegend	Cat#121104; RRID: AB_493502
Anti-mouse CD62L (MEL-14)	Biolegend	Cat#104438; RRID: AB_2563058
Anti-mouse CD117 (c-Kit) (2B8)	Biolegend	Cat#105824; RRID: AB_2131597
Anti-mouse IL-5 (TRFK5)	Biolegend	Cat#504306; RRID: AB_315330
Anti-mouse CCR6 (CD196) (29-2L17)	Biolegend	Cat#129814; RRID: AB_1877147
Anti-mouse CD61 (HMβ3-1)	Biolegend	Cat#13-0611-81; RRID: AB_466487
Anti-mouse I-A/I-E (M5/144.15.2)	Biolegend	Cat#107636; RRID: AB_2734168
Anti-human CD2 (RPA-2.10)	Biolegend	Cat#300218; RRID: AB_2566040
Anti-mouse CD45 (30-F11)	eBioscience	Cat#56-0451-82; RRID: AB_891454
Anti-mouse CD45.2 (104)	eBioscience	Cat#56-0454-82; RRID: AB_657752
Anti-mouse KLRG1 (2F1)	eBioscience	Cat#46-5893-82; RRID: AB_10670282
Anti-mouse CD49b (DX5)	eBioscience	Cat#17-5971-82; RRID: AB_469485
Anti-mouse CD44 (IM7)	eBioscience	Cat#17-0441-82; RRID: AB_469390
Anti-mouse α4β7 Integrin (DATK32)	eBioscience	Cat#46-5887-82; RRID: AB_2573793
Anti-mouse PD-1 (J43)	BD Biosciences	Cat#565815; RRID: AB_2739366
Anti-mouse Sca-1(D7)	eBioscience	Cat#45-5981-82; RRID: AB_914372
Anti-mouse IL-13 (eBio13A)	eBioscience	Cat#12-7133-82; RRID: AB_763559
Anti-mouse CD200R (OX-110)	eBioscience	Cat#46-5201-82; RRID: AB_10804765
Anti-mouse CD25 (PC61.5)	eBioscience	Cat# 46-0251-82; RRID: AB_2734935
Anti-mouse Flt3 (CD135) (A2F10)	eBioscience	Cat#46-1351-82; RRID: AB_10733393
Anti-mouse Eomes (Dan 11mag)	eBioscience	Cat#46-4875-82; RRID: AB_10597455
Anti-mouse Tbet (eBio4B10)	eBioscience	Cat#50-5825-82; RRID: AB_10596655
Anti-mouse Perforin (S16009B)	Biolegend	Cat#154404; RRID: AB_2721465
Anti-mouse CD49a (Ha31/8)	BD Biosciences	Cat#564862; RRID: AB_2734135)
Anti-mouse T1/ST2 (DJ8)	MD Bioproducts	Cat#101001B; RRID: AB_947551
Anti-human/mouse/rat PLZF (D-9)	Santa Cruz	Cat#sc-28319; RRID: AB_2218941

**Chemicals, Peptides, and Recombinant Proteins**		

Collagenase I	GIBCO	Cat#17100-017
DNase I, from bovine pancreas	Sigma-Aldrich	Cat#D5025/DN25
Fixable Viability Dye eFluor780	Invitrogen	Cat#65-0865-14
Liberase TL, research grade	Roche	Cat#385040
PBS (endotoxin-free)	Sigma-Aldrich	Cat#D1408
Fetal Calf Serum	GIBCO	Cat#10270-106
Percoll	GE Healthcare	Cat#17-0891-01
HEPES	GIBCO	Cat#15630-056
2-Mercaptoethanol	Sigma-Aldrich	Cat#M6250
RPMI 1640 + GlutaMAX	GIBCO	Cat#61870-010
IMDM	GIBCO	Cat#31980-022
Non-EAA	GIBCO	Cat#111040-050
Protein Transport Inhibitor Cocktail	Invitrogen	Cat#00-4980-93
GolgiPlug™	BD Biosciences	Cat#51-2301KZ
16% Paraformaldehyde, Methanol-free	ThermoScientific	Cat#28906
Foxp3 Staining Kit	eBioscience	Cat#00-5523-00
Cytofix/Cytoperm Plus Kit	BD Biosciences	Cat#555028
PMA (phorbol 12-myristate 13-acetate)	Sigma-Aldrich	Cat#P8139
Ionomycin	Sigma-Aldrich	Cat#I0634
rmIL-7, carrier-free	Biolegend	Cat#577806
rmSCF, carrier-free	Biolegend	Cat#579706
rmIL-2, carrier-free	Biolegend	Cat#575406
rmIL-15, carrier-free	Biolegend	Cat#566304
rmIL-18, carrier-free	Biolegend	Cat#767006
Agencourt AMPure XP beads	Beckman Coulter	Cat#A63881

**Critical Commercial Assays**		

Foxp3 staining kit	eBioscience	Cat#00-5523-00
Illumina Nextera XT DNA Library Preparation Kit	Illumina	Cat#C-131-1096
Qubit dsDNA HS assay kit	Invitrogen	Cat#Q32851

**Deposited Data**		

Single-cell RNA sequencing data	This paper	GEO accession number GSE131038

**Experimental Models: Cell Lines**		

JM8 ES cells	Welcome Sanger Institute	N/A
Mouse: OP9	Sunnybrook Research Institute	Schmitt and Zúñiga-Pflücker, 2002
Mouse: OP9-DL1	Sunnybrook Research Institute	Schmitt and Zúñiga-Pflücker, 2002

**Experimental Models: Organisms/Strains**		

Mouse: C57BL/6JOla	Jackson Labs (Bred in LMB)	Cat#000664; RRID: IMSR_JAX:000664
Mouse: CD45.1 *Rag2*^−/−^*Il2rgc*^−/−^	Institute Pasteur	Serafini et al., 2014
Mouse: *Bcl11b*^*+/tdTom*^	Sanger Institute	Li et al., 2010
Mouse: *Rorc*^*+/Kat*^	MRC-LMB	N/A
Mouse: *Id2*^*+/BFP*^	MRC-LMB	N/A
Mouse: *Gata3*^*+/hCD2*^	MRC-LMB	N/A
Mouse: *Rora*^*+/Teal*^	MRC-LMB	N/A
Mouse: *Zbtb16*^*tdTom*^	MRC-LMB	N/A
Mouse: *Id2*^*+/BFP*^*Gata3*^*+/hCD2*^*Rora*^*+/Teal*^*Bcl11b*^*+/tdTom*^ (4x polychromILC)	MRC-LMB	N/A
Mouse: *Id2*^*+/BFP*^*Gata3*^*+/hCD2*^*Rora*^*+/Teal*^*Bcl11b*^*+/tdTom*^*Rorc*^*+/Kat*^ (5x polychromILC)	MRC-LMB	N/A

**Oligonucleotides**		

Primer: *Id2* target verification: F1 AAATGGG GGGCGTCCCAGTAGGTAGCTGGGGTGGCC (5′-3′)	Sigma-Aldrich	Custom made
Primer: *Id2* target verification: R1 CTCGGATGTGCACTTGAAGTGATGGTTGTC CACGGTGCCC (5′-3′)	Sigma-Aldrich	Custom made
Primer: *Id2* target verification: F2 CCCTTCACA CCTTCCCACCACCCACGGATCCCGCCC (5′-3′)	Sigma-Aldrich	Custom made
Primer: *Id2* target verification: R2 TACAGCTTCA TGTGCATGTTCTCCTTAATCAGCTCGC (5′-3′)	Sigma-Aldrich	Custom made
Primer: *Gata3* targeting verification: F1 CTGTTGACAATTAATCATCGGCATAGTATATCGGC (5′-3′)	Sigma-Aldrich	Custom made
Primer: *Gata3* targeting verification: R1 CTCCTCC TCCTTCTTCATCATCAAAAGAGCC (5′-3′)	Sigma-Aldrich	Custom made
Primer: *Zbtb16* targeting verification: F1 AGAAGACAGGGTGCTTATGGCTGACACGTGA GTGGC (5′-3′)	Sigma-Aldrich	Custom made
Primer: *Zbtb16* targeting verification: R1 CTAAAGCGCATGCTCCAGACTGCCTTGGGAA AAGCG (5′-3′)	Sigma-Aldrich	Custom made
Primer: *Zbtb16* targeting verification: F2 CCCGTGCCTTCCTTGACCCTGGAAGGTGCC ACTCCC (5′-3′)	Sigma-Aldrich	Custom made
Primer: *Zbtb16* targeting verification: R2 GGAGG GCCTCTACCAGGTCAAGTTCAAAGCC (5′-3′)	Sigma-Aldrich	Custom made
Primer: *Zbtb16* guide RNA: G1 CCCAACACATG GTAGAGCAG**TGG** (5′-3′)	Sigma-Aldrich	Custom made
Primer: *Zbtb16* guide RNA: G2 CCCCCCTTCAC ACATAACAC AGG (5′-3′)	Sigma-Aldrich	Custom made
Primer: *Rorc*(γt)^Kat^ genotyping: Kat F: CTGAGAG GCCATAGCCAGATG (5′-3′)	Sigma-Aldrich	Custom made
Primer: *Rorc*(γt)^Kat^ genotyping: Kat R: TTAGCGG GTTTCTTGGATCTGT (5′-3′)	Sigma-Aldrich	Custom made
Primer: *Rorc*(γt)^Kat^ genotyping: Kat Probe: FAM CTGCACTGCTCCCTCAAGACCAC TAMRA (5′-3′)	Sigma-Aldrich	Custom made
Primer: *Rorc*(γt)^Kat^ genotyping: Rorc kat WT F: GGGAGCCAAGTTCTCAGTCATG (5′-3′)	Sigma-Aldrich	Custom made
Primer: *Rorc*(γt)^Kat^ genotyping: Rorc kat WT R: CGGTTTCCAAGATACACTCCTATTC (5′-3′)	Sigma-Aldrich	Custom made
Primer: *Rorc*(γt)^Kat^ genotyping: Rorc kat WT probe: VIC TCCTGTCACCATTCCTAGGCCCGC TAMRA (5′-3′)	Sigma-Aldrich	Custom made
Primer: *Gata3*^hCD2^ genotyping: hCD2 WT F: CACCGC CATGGGTTAGAGA (5′-3′)	Sigma-Aldrich	Custom made
Primer: *Gata3*^hCD2^ genotyping: hCD2 WT R: ATACTGC TCCTGCGAAAAACG (5′-3′)	Sigma-Aldrich	Custom made
Primer: *Gata3*^hCD2^ genotyping: hCD2 WT Probe: VIC CTCCACATGCGTGAGGAGTCTCCA TAMRA (5′-3′)	Sigma-Aldrich	Custom made
Primer: *Gata3*^hCD2^ genotyping: hCD2 Mut F: TGGAGA GGGCAGAGGAAGTCT (5′-3′)	Sigma-Aldrich	Custom made
Primer: *Gata3*^hCD2^ genotyping: hCD2 Mut R: AATCAG AAGGAAGCTGGCTACAA (5′-3′)	Sigma-Aldrich	Custom made
Primer: *Gata3*^hCD2^ genotyping: hCD2 Mut Probe: FAM AGAATCCTGGCCCAATGAGCTTTCCA TAMRA (5′-3′)	Sigma-Aldrich	Custom made
Primer: *Rora*^Teal^ genotyping: Rora teal WT F: GCCAGC CATGCAAATCG (5′-3′)	Sigma-Aldrich	Custom made
Primer: *Rora*^Teal^ genotyping: Rora teal WT R: TCTTCG TTGTTATTGTTTCATTTCCT (5′-3′)	Sigma-Aldrich	Custom made
Primer: *Rora*^Teal^ genotyping: Rora teal WT Probe: VIC ATGTCGCGCCCGAGCACTTC TAMRA (5′-3′)	Sigma-Aldrich	Custom made
Primer: *Rora*^Teal^ genotyping: Teal F: CCGACGACATCC CCAACTAC (5′-3′)	Sigma-Aldrich	Custom made
Primer: *Rora*^Teal^ genotyping: Teal R: TGGTGCGCTCC CAAGAGT (5′-3′)	Sigma-Aldrich	Custom made
Primer: *Rora*^Teal^ genotyping: Teal Probe: FAM CAAGC AGTCCTTCCCCGAGGGC TAMRA (5′-3′)	Sigma-Aldrich	Custom made
Primer: *Id2*^BFP^ genotyping: Id2 WT F: TCCCTTCTGAG CTTATGTCGAAT (5′-3′)	Sigma-Aldrich	Custom made
Primer: *Id2*^BFP^ genotyping: Id2 WT R: AACATTTAACA GACACACAAGCACATT (5′-3′)	Sigma-Aldrich	Custom made
Primer: *Id2*^BFP^ genotyping: Id2 Probe: VIC CCT CCT GTG TGC GCG TTT CGG TAMRA (5′-3′)	Sigma-Aldrich	Custom made
Primer: *Id2*^BFP^ genotyping: Id2 BFP F: TGGAAGGCAG AAACGACATG (5′-3′)	Sigma-Aldrich	Custom made
Primer: *Id2*^BFP^ genotyping: Id2 BFP R: GGTTTCTTGGA TCTATATGTGGTCTTG (5′-3′)	Sigma-Aldrich	Custom made
Primer: *Id2*^BFP^ genotyping: Id2 BFP Probe: FAM CGGGA GCCATCTGTTCGCAAAC TAMRA (5′-3′)	Sigma-Aldrich	Custom made

**Software and Algorithms**		

Prism 7	GraphPad Prism	RRID: SCR_002798
FlowJo	FlowJo, LLC	v10, RRID: SCR_008520
R	R Foundation for StatisticalComputing, Vienna, Austria	v3.4.1
Salmon pseudoaligner	https://salmon.readthedocs.io/en/latest/salmon.html Patro et al., 2017	v0.8.2
Scater library	https://bioconductor.org/packages/release/bioc/html/scater.html Lun et al., 2016	v1.6.3
Surrogate Variable Analysis library	https://bioconductor.org/packages/release/bioc/html/sva.html	R package v3.26.0
Scran library	http://bioconductor.org/packages/release/bioc/html/scran.html Lun et al., 2016	V3.8
Monocle library	Qiu et al., 2017a, Qiu et al., 2017b, Trapnell et al., 2014	V2.6.4

**Other**		

Sony Biotechnology SY3200	N/A	N/A
BD LSRFortessa Special Order (5 laser)	N/A	N/A

### Contact for Reagents and Resource Sharing

Correspondence and request for materials should be addressed to Andrew McKenzie (anm@mrc-lmb.cam.ac.uk)

### Method Details

#### Mice

All mice were bred in a specific pathogen-free facility. In individual experiments, mice were matched for age, sex and background strain and all experiments undertaken in this study were done so with the approval of the UK Home Office. Bcl11b^tdTom^ mice were provided by Pentao Liu (Wellcome Sanger Institute, Cambridge, UK), CD45.1 *Rag2*^*−/−*^*Il2rgc*^*−/−*^ mice were a gift from James Di Santo and C57BL/6JOla mice were maintained in house.

#### Generation of *Rorc*^Kat^ gene-targeted mice

*Rorc*^Kat^ mice were generated according to the strategy shown in Figure S1A. Briefly, the gene encoding Katushka fluorescent protein, followed by a loxP-flanked Neomcyin cassette, was inserted directly downstream of the ATG start codon for the *Rorc* transcript. Successful targeting of JM8 ES cells was confirmed by Southern blot (Figure S1B) and the neomycin cassette was removed from the resultant mice by inter-crossing with a Cre recombinase strain. Genotyping was performed by quantitative PCR (see ‘Primers’).

#### Generation of *Id2*^BFP^ gene-targeted mice

The *Id2* gene was targeted by standard homologous recombination in mouse JM8 ES cells. Appropriate homologous recombination of the *Id2*-blue fluorescent protein targeting construct was confirmed at the 3′ end by Southern blotting (Figures S2A and S2B). At the 5′ end nested PCR using primers F1 and R1 followed by F2 and R2 was used to amplify a fragment which spanned from within the tagBFP sequence to beyond the end of the 5′ arm of homology. This fragment was then sequence verified. Genotyping was performed by quantitative PCR (see ‘Primers’).

#### Generation of *Gata3*^hCD2^ gene-targeted mice

The *Gata3* was targeted by standard homologous recombination in mouse JM8 ES cells. Appropriate homologous recombination of the *Gata3*-human CD2 targeting construct was confirmed at the 5′ end by Southern blot analysis (Figures S3A and S3B). At the 3′ side PCR using primers F1 and R1 was used to amplify a fragment which spanned from within the PGK-Neo cassette to beyond the end of the 3′ arm of homology. This fragment was then sequence verified. Genotyping was performed by quantitative PCR (see ‘Primers’).

#### Generation of *Rora*^Teal^ gene-targeted mice

*Rora*^Teal^ mice were generated according to the strategy shown in Figure S4A. Briefly, a reporter cassette, encoding a short Gly-Ser-Gly linker peptide, FLAG epitope tag, T2A self-cleaving peptide and Teal fluorescent protein, followed by a loxP-flanked neomcyin cassette, was inserted (directly upstream of the) *Rora* stop codon. Successful targeting of JM8 ES cells was confirmed by Southern blot analysis (Figure S4B) and the neomycin cassette was removed from the resultant mice by inter-crossing with a Cre recombinase strain. Genotyping was performed by quantitative PCR (see ‘Primers’).

#### Generation of *Zbtb16*^tdTom^ gene-targeted mice

The *Zbtb16* gene was targeted using CRISPR technology. Expression constructs encoding two guide RNA’s of opposing orientation (G1 and G2) and the D10A nickase mutant of Cas9 were cotransfected into JM8 ES cells with the targeting construct shown in (Figure S5A), to mediate insertion of the cassette by homology directed repair. Appropriate insertion was confirmed at both the 5′ and 3′ ends by PCR amplification of fragments spanning from within the PGK-Neo cassette to beyond the ends of the respective arms of homology using primers F1 and R1 (5′side) and F2 and R2 (3′side). These fragments were then sequence verified.

#### Tissue preparation

Cell suspensions of spleen, MLN, liver and thymus were obtained by passing the tissues through a 70 μm strainer. Lung tissue was pre-digested with 750 U/mL collagenase I (GIBCO) and 0.3 mg/mL DNaseI (Sigma-Aldrich) prior to obtaining a single cell suspension. Bone marrow was removed from femurs, tibiae and hips by flushing with PBS + 2% FCS, or by centrifuging briefly at 6000 x g. For bone marrow, lung, liver and spleen cell suspensions, red blood cells were removed by incubation with RBC lysis solution (140 mM NH_4_Cl, 17 mM Tris; pH 7.2). Lung lymphocytes were further enriched by centrifugation in 30% percoll at 800 x g (GE Healthcare) while liver lymphocytes were enriched in 40% percoll at 690 x g.

For preparation of siLP lymphocytes, intestinal contents were removed by the application of gentle pressure along the length of the intestine. Intestines were opened longitudinally, cut into 3 cm long pieces and washed briefly by vortexing in PBS + 10 mM HEPES (PBS/HEPES). Epithelial cells were removed by incubation with RPMI supplemented with 2% FCS, 1 mM dithiothreitol and 5 mM EDTA for 2 × 20 min at 37°C with shaking (200 rpm). Intestinal pieces were washed with PBS/HEPES and incubated, with shaking, at 37°C with RPMI + 2% FCS, 0.125 KU/mL DNaseI (Sigma-Aldrich) and 62.5 μg/mL Liberase TL (Roche) until no large pieces of intestine remained. Cells were then passed through a 70 μm strainer, pelleted and separated over a 40%:80% gradient of Percoll at 600 x g for 20 min. siLP lymphocytes were isolated from the interface and prepared for flow cytometric analysis. Unless stated otherwise, small intestine lamina propria (siLP) includes associated Peyer’s patches.

#### Flow cytometry

Single cell suspensions were incubated with fluorochrome-, or biotin-, conjugated antibodies in the presence of anti-CD16/CD32 antibody (Fc block, clone 2.4G2), followed by fluorochrome-conjugated streptavidin where necessary. Antibodies were from Biolegend (CD3e (145-2C11), CD11b (M1/70), IL-7Rα (SB/199), CD62L (MEL-14), c-Kit (2B8), IL-5 (TRFK5), CCR6 (29-2L17), CD61 (HMβ3-1), MHCII (I-A/I-E, M5/144.15.2)); eBioscience (hCD2 (RPA-2.10), CD4 (GK1.5), CD5 (53-7.3), CD8 (53-6.7), CD19 (eBio1D3), NK1.1 (PK136), CD11c (N418), Gr-1 (RB6-8C5), FcεRI (MAR-1), Ter-119 (TER-119), CD45 (30-F11), KLRG1 (2F1), NKp46 (29A1.4), CD49b (DX5), CD44 (IM7), α4β7 integrin (DATK32), PD-1 (J43), Sca-1 (D7), IL-13 (eBio13A), CD200R1 (OX-110), CD25 (PC61.5), Flt3 (A2F10), Eomes (Dan11mag), Tbet (eBio4B10), perforin (S16009B), IFNγ (XMG1.2)); BD Biosciences (CD49a (Ha31/8)); MD Bioproducts (ST2 (DJ8)) and Santa Cruz (PLZF (D-9)). ‘Lineage’ staining included antibodies specific for CD3, CD4, CD5, CD8, CD11b, CD11c, CD19, FcεRI, Gr-1, NK1.1 and Ter-119 (although in some instances individual lineage markers were analyzed in separate channels, as indicated in figures). All samples were co-stained with a cell viability dye (Fixable dye eFluor780, Invitrogen) and analysis was performed on an LSRFortessa system (BD Biosciences). For cell sorting an iCyt Synergy (70 μm nozzle, Sony Biotechnology) was used. Intracellular transcription factor staining was performed by fixation with 2% PFA for 45 min, followed by incubation with fluorochrome antibodies diluted in perm wash buffer (Foxp3 staining kit, eBioscience). Intracellular cytokine was performed using BD Cytofix/Cytoperm Plus reagents (BD Biosciences) following pre-culture with RPMI, supplemented with 50 ng/mL phorbol 12-myristate 13-acetate (PMA), 500 ng/mL ionomycin and protein transport inhibitor (GolgiPlug®, BD Biosciences), for 4 h at 37°C.

#### Adoptive transfers

Populations of mouse bone marrow cells were FACS purified into heat-inactivated mouse serum, diluted to 50% with PBS. Cell suspensions were aspirated with a syringe and implanted via tail vein injection into sublethally-irradiated (450 rad) *Rag2*^−/−^*Il2rgc*^−/−^ recipients. Analysis of donor cell progeny was performed 5 to 6 weeks after cell transfer.

#### OP9 stromal cell co-cultures

OP9 cells were maintained in complete IMDM (IMDM, supplemented with 20% FCS, 1% penicillin, 1% streptomycin, 0.1% 2-mercaptoethanol and non-essential amino acids (GIBCO)). For progenitor cell co-culture, OP9 cells were incubated with 4 μg/mL mitomycin C for 2 h, washed, seeded at a density of 1 × 10^6^ cells per 96-well plate and allowed time to adhere. Sorted ILC progenitor populations were seeded onto OP9 monolayers and cultured in complete IMDM, supplemented with 25 ng/mL rmIL-7 (Biolegend) and 25 ng/mL rmSCF (Biolegend), for 7 days before flow cytometric analysis of progeny. For clonal analysis of progenitor cell potential, single cells were sorted directly onto OP9 monolayers and cultured in the presence of 25 ng/mL rmIL-7 and 25 ng/mL rmSCF for 14 - 19 days. Flow cytometric analysis was performed and wells were considered to be positive for a cell population if > 3 cells appeared within the relevant gate. For analysis of perforin and IFN-γ expression cell subsets were seeded onto OP9 monolayers and cultured in complete IMDM, supplemented with 25 ng/mL rmIL-7 (Biolegend) and 25 ng/mL rmSCF (Biolegend), for 7 days and then stimulated with IL-2, IL-15 and IL-18 (all at 50 ng/mL) for 48 h before flow-cytometric analysis.

#### Single cell RNA-sequencing and analysis

Single cell RNaseq libraries were prepared essentially as described previously (Picelli et al., 2014), with modifications as described below. Individual cells were flow cytometrically purified on a 96 well format into 0.2% Triton X-100 containing RNase inhibitor, dNTPs and oligo-dT primers and stored at −80°C. On thawing, lysates were heated to 72°C for 3 min and subject to reverse transcription, PCR preamplification (26 cycles) and PCR purification. cDNA library quality was assessed for all samples by qualitative PCR using primers for 18 s RNA with an additional check by Bioanalyzer using an Agilent high sensitivity DNA chip on a small subset of libraries. A subset of libraries was quantified using the Qubit dsDNA HS assay kit and an average value used to calculate library dilution to 100-150 pg/μl.

cDNA library tagmentation and amplification was performed using the Illumina Nextera XT DNA Library Preparation Kit according to manufacturer’s instructions (except that all volumes were reduced to 25% of recommended volumes) and tagmentation performed at 55°C for 20 min. Nextera index and Illumina adaptor sequences were incorporated at the amplification stage (N7xx and S5xx). Amplified and indexed libraries were pooled and purified using Agencourt AMPure XP beads at a ratio of 1:0.9 library to beads and washed with 70% ethanol. Two rounds of purification were performed before a final elution in 1.25x total library volume of Nextera Resuspension buffer. Pooled indexed libraries were quantified using the Qubit dsDNA HS assay kit and this was confirmed by qPCR with adaptor specific primers. Quality was assessed by Bioanalyzer using an Agilent high sensitivity DNA chip. All pooled libraries had an average fragment length of between 500 and 800bp. Libraries were sequenced at the CRUK Cambridge NGS facility.

Reads were aligned to a modified mouse genome (v38.68), which had the 92 ERCC spike-ins added, using the Salmon pseudoaligner (v0.8.2) (Patro et al., 2017). The resulting pseudocounts were then analyzed using R (v3.4.1) (https://www.R-project.org/) and the scater library (v1.6.3) (McCarthy et al., 2017), scran library (v1.6.9) (Lun et al., 2016), and sva library (sva: Surrogate Variable Analysis. R package v3.26.0). Cells with pseudocounts below 3 median-absolute-deviations away from the median were removed. The same threshold was applied to number of genes detected, percentage of counts mapping to mitochondrial genes and percentage of counts mapping to spike-ins. Genes with an average count across all remaining cells of less than 1 were removed. Size factors were then calculated using the scran library (based on the gene counts) and the data normalized by them, as described previously (Lun et al., 2016). Finally, the batch effects caused by the use of different sequencing facilities were removed using the ComBat empirical Bayes framework from the sva library.

Highly variable genes were identified using the scran library, as described, with Local Polynomial Regression Fitting (loess) and alpha = 0.4. Only genes with FDR ≤ 0.05 and biological component of the variance > = 0.5 were considered to be highly variable. Hierarchichal clustering was performed, based on the expression of these genes, using the hclust function with the ward.D2 method. Differential expression of genes between the clusters was identified using the findMarkers function from the scran library.

A heatmap was compiled for the expression of selected genes in those bone marrow cells that lacked Spi1, Sox13, or Gata1. Tree building was performed using the hclust function with the average method.

Pseudotime analysis was performed using the monocle library (v2.6.4) (Qiu et al., 2017a, Qiu et al., 2017b, Trapnell et al., 2014). Only those bone marrow cells that did not express Spi1, Sox13, or Gata1 and that were sequenced at the CRUK (greater read depth) were selected (i.e., clusters C1-C3). Analysis was performed as described in the library documentation, with rho_threshold = 2 and delta_threshold = 8 for clustering.

#### Statistical analysis

Statistical analysis was performed using GraphPad Prism v7.0b software.

### Data and Software Availability

Single-cell RNA sequencing data have been deposited in the Gene Expression Omnibus under accession number GEO: GSE131038.

## supplemental Information

Document S1. Figures S1–S7 and Table S1

Document S2. Article plus Supplemental Information
