# Supplementary material for: Polychromic Reporter Mice Reveal Unappreciated Innate Lymphoid Cell Progenitor Heterogeneity and Elusive ILC3 Progenitors in Bone Marrow
Source: Immunity. 2019 Jul 16;51(1):104–118.e7. doi: 10.1016/j.immuni.2019.05.002 (PMC6642165; doi:10.1016/j.immuni.2019.05.002)
Supplement: Document S1. Figures S1–S7 and Table S1 [file mmc1.pdf]

Immunity, Volume *51*

Supplemental Information

Polychromic Reporter Mice Reveal Unappreciated Innate Lymphoid Cell Progenitor Heterogeneity and Elusive ILC3 Progenitors in Bone Marrow

Jennifer A. Walker, Paula A. Clark, Alastair Crisp, Jillian L. Barlow, Aydan Szeto, Ana C.F. Ferreira, Batika M.J. Rana, Helen E. Jolin, Noe Rodriguez-Rodriguez, Meera Sivasubramaniam, Richard Pannell, James Cruickshank, Maria Daly, Liora Haim-Vilmovsky, Sarah A. Teichmann, and Andrew N.J. McKenzie

SUPPLEMENTAL INFORMATION

Figure S1. Generation and immune phenotyping of *Rorc*^{Kat} mice. Related to Figure 1.
(A) Gene-targeting strategy used to generate the *Rorc*^{Kat} strain (WT = wildtype). (B) Southern blot analysis of *EcoRV*-digested (left) and *HindIII*-digested (right) genomic DNA from WT and *Rorc*$^{Kat/+}$ mice (TG = Transgenic). (C) Frequency of the indicated ILC populations in the siLP of *Rorc*^{Kat} littermates (gated as in Figure 1A). (D) Gating of thymocyte populations in *Rorc*^{Kat} littermates. (E) Frequency of thymocyte populations in *Rorc*^{Kat} littermates (DP = CD4^{+}CD8^{+}, CD4SP = CD4^{+}CD8^{-}, CD8SP = CD4^{-}CD8^{+}). (F) Rorc-Kat fluorescence of DP thymocytes from the indicated *Rorc*^{Kat} littermates. (G) Rorc-Kat fluorescence of CD45^{+}Lin^{-}IL-7Rα^{+}CD4^{-}KLRG1^{-} NKp46^{+}NK1.1^{+} ILC3s in the siLP of *Rorc*^{Kat} littermates. Data are representative of 2 independent experiments with 3 - 4 mice per group. Data represent mean ± SEM; not significant (ns); one-way ANOVA with *Dunnett's* post-hoc test.

Figure S2. Generation and immune phenotyping of *Id2*^{BFP} mice. Related to Figure 1.
(A) Gene-targeting strategy to generate the *Id2*^{BFP} strain and location of nested primers (F1, R1, F2 and R2) used for sequence verification of gene-targeting (WT = wildtype). (B) Southern blot analysis of *KpnI*-digested genomic DNA from wildtype and heterozygous *Id2*$^{BFP/+}$ mice (TG = Transgenic). (C) Id2-BFP fluorescence of splenic NK cells (CD45^{+}CD3^{-}CD19^{-} NK1.1^{+}NKp46^{+}) from I*d2*^{BFP} littermates. (D) Frequency of the indicated ILC populations in the siLP of *Id2*^{BFP} littermates (gated as in Figure 1A). (E) Frequency of CD11c^{+}MHCII^{+} dendritic cells (DC) in the spleen of *Id2*^{BFP} littermates. (F) Frequency of the indicated splenic lymphocytes in *Id2*^{BFP} littermates (naïve CD4+ = CD45^{+}CD3^{+}CD4^{+}CD62L^{hi}CD44^{lo}; activated CD4+ = CD45^{+}CD3^{+}CD4^{+}CD62L^{lo}CD44^{hi}; CD25+CD4+ = CD45^{+}CD3^{+}CD4^{+}CD25^{+}; CD8+ = CD45^{+}CD3^{+}CD8^{+}, NK1.1^{+}NKp46^{+} = CD45^{+}CD3^{-}CD19^{-}NK1.1^{+}NKp46^{+}). Data are representative of 2 independent experiments with 4 - 5 mice per group. Data represent mean ± SEM; not significant (ns); one-way ANOVA with *Dunnett's* post-hoc test.

Figure S3. Generation and immune phenotyping of *Gata3*^{hCD2} mice. Related to Figure 1
(A) Gene-targeting strategy to generate the *Gata3*^{hCD2} strain and location of forward (F) and reverse (R) primers used for sequence verification of gene-targeting (WT = wildtype). (B) Southern blot analysis of *KpnI*-digested genomic DNA from wildtype and heterozygous *Gata3*^{hCD2} mice (TG = Transgenic). Tracks either side of the dashed line were not adjacent on the original autoradiograph. (C) Gata3-hCD2 expression in ILC2 (CD45^{+}Lin^{-}IL-7Rα^{+}KLRG1^{+}) from the mesenteric lymph node (MLN) of *Gata3*^{hCD2} littermates following intraperitoneal (i.p.) injection of PBS or 0.5 μg IL-33 i.p on 3 consecutive days. (D) Numbers of ILC2 (CD45^{+}Lin^{-}

IL-7Rα$^+$KLRG1$^+$) in the MLN of *Gata3*^{hCD2} littermates and C57Bl/6 controls following i.p. injection of PBS or 0.5 μg IL-33 on 3 consecutive days. (E) Frequency of cytokine-producing ILC2 (CD45$^+$Lin$^-$IL-7Rα$^+$KLRG1$^+$) in the MLN of *Gata3*^{hCD2} littermates and C57Bl/6 controls following i.p. injection of 0.5 μg IL-33 on 3 consecutive days. (F) Frequency of ILC2 (CD45$^+$Lin$^-$ IL-7Rα$^+$CD25$^+$ST2$^+$) in lung from *Gata3*^{hCD2} littermates and C57Bl/6 controls. (G) Frequency of ILC2P (CD45$^+$Lin$^-$IL-7Rα$^+$Flt3$^-$Sca-1$^+$α4β7^{int}ST2$^+$) in bone marrow from *Gata3*^{hCD2} littermates and C57Bl/6 controls. (H) Frequency of the indicated ILC populations in the siLP of *Gata3*^{hCD2} littermates (gated as in Figure 1A). (I) Frequency of the indicated splenic lymphocytes in *Gata3*^{hCD2} littermates (naïve CD4+ = CD45$^+$CD3$^+$CD4$^+$CD62L^{hi}CD44^{lo}; activated CD4+ = CD45$^+$CD3$^+$CD4$^+$CD62L^{lo}CD44^{hi}; CD25+CD4+ = CD45$^+$CD3$^+$CD4$^+$CD25$^+$; CD8+ = CD45$^+$CD3$^+$CD8$^+$, NK1.1$^+$NKp46$^+$ = CD45$^+$CD3$^-$CD19$^-$NK1.1$^+$NKp46$^+$). Data are representative of two independent experiments with 3 - 6 mice per group. Data represent mean ± SEM; not significant (ns); one-way ANOVA with *Dunnett's* post-hoc test.

Figure S4. Generation and immune phenotyping of *Rora*^{Teal} mice. Related to Figure 1
(A) Gene-targeting strategy used to generate the *Rora*^{Teal} strain (WT = wildtype). (B) Southern blot analysis of *Nco1*-digested genomic DNA from wildtype and heterozygous *Rora*^{Teal} mice (TG = Transgenic). Tracks either side of the dashed line were not adjacent on the original autoradiograph. (C) Rora-Teal expression in ILC2 (CD45$^+$Lin$^-$IL-7Rα$^+$KLRG1$^+$) from the MLN of *Rora*^{Teal} littermates and C57Bl/6 controls following i.p. injection of PBS or 0.5 μg IL-33 i.p on 3 consecutive days. (D) Rora-Teal expression in ILC2P (CD45$^+$Lin$^-$IL-7Rα$^+$ST2$^+$) from the bone marrow of *Rora*^{Teal} littermates and C57Bl/6 controls. (E) Numbers of ILC2 (CD45$^+$Lin$^-$IL-7Rα$^+$KLRG1$^+$) in the MLN of *Rora*^{Teal} littermates and C57Bl/6 controls following i.p. injection of PBS or 0.5 μg IL-33 on 3 consecutive days. (F) Frequency of cytokine-producing ILC2 (CD45$^+$Lin$^-$IL-7Rα$^+$KLRG1$^+$) in the MLN of *Rora*^{Teal} littermates and C57Bl/6 controls following i.p. injection of 0.5 μg IL-33 on 3 consecutive days. (G) Frequency of ILC2 (CD45$^+$Lin$^-$IL-7Rα$^+$CD25$^+$ST2$^+$) in lung from *Rora*^{Teal} littermates and C57Bl/6 controls. (H) Frequency of ILC2P (CD45$^+$Lin$^-$IL-7Rα$^+$Flt3$^-$Sca-1$^+$α4β7^{int}ST2$^+$) in bone marrow from *Rora*^{Teal} littermates and C57Bl/6 controls. (I) Frequency of the indicated ILC populations in the siLP of *Rora*^{Teal} littermates and C57Bl/6 controls (gated as described in Figure 1A). (J) Frequency of the indicated splenic lymphocytes in *Rora*^{Teal} littermates and C57Bl/6 controls (naïve CD4+ = CD45$^+$CD3$^+$CD4$^+$CD62L^{hi}CD44^{lo}; activated CD4+ = CD45$^+$CD3$^+$CD4$^+$CD62L^{lo}CD44^{hi}; CD25+CD4+ = CD45$^+$CD3$^+$CD4$^+$CD25$^+$; CD8+ = CD45$^+$CD3$^+$CD8$^+$, NK1.1$^+$NKp46$^+$ = CD45$^+$CD3$^-$CD19$^-$NK1.1$^+$NKp46$^+$). (K) Flow cytometry gating strategy for intracellular TF staining of Lin$^-$NK1.1$^+$ cells from the indicated tissues of *Rora*$^{+/Teal}$ mice, compared to C57Bl/6

controls. Data are pooled from two independent experiments with 3 - 5 mice per group. Data represent mean ± SEM; not significant (ns); one-way ANOVA with *Dunnett's post*-hoc test.

Figure S5. Generation of *Zbtb16*^{tdTom} mice. Related to Figure 5

(A) Gene-targeting strategy used to generate the *Zbtb16*^{tdTom} strain and location of primers (F1, R1, F2 and R2) used for sequence verification of gene-targeting (WT = wildtype). (B) Southern blot analysis of *HinDIII*-digested genomic DNA from *Zbtb16*^{tdTom} targeted ES cell clone using 5' probe indicated in (A). (C) Zbtb16-tdTom expression in NKT cells ($CD4^{+}TCR\beta^{lo}NK1.1^{+}$) from the liver of *Zbtb16*$^{+/tdTom}$ and WT littermates compared to intracellular staining for PLZF. (D) Frequency of CLP ($Lin^{-}CD45^{+}$ $IL\text{-}7R\alpha^{+}Flt3^{+}$), ILC2P ($Lin^{-}CD45^{+}IL\text{-}7R\alpha^{+}\alpha4\beta7^{+}Sca1^{hi}Flt3^{-}CD25^{+}$) CHILP ($Lin^{-}CD45^{+}IL\text{-}7R\alpha^{+}\alpha4\beta7^{hi}Sca1^{int/lo}Flt3^{-}CD25^{-}$), and HSC ($Lin^{-}CD45^{+}Kit^{+}Sca1^{+}Flt3^{-}$) in bone marrow from *Zbtb16*$^{+/tdTom}$ and *Zbtb16*$^{tdTom/tdTom}$ littermates and C57Bl/6 controls. Data representative of two independent experiments with 4 - 8 mice per genotype. (E) Frequency of the indicated ILCs from *Zbtb16*$^{+/tdTom}$ and *Zbtb16*$^{tdTom/tdTom}$ littermates and C57Bl/6 controls (gated as in Figure 1A) in siLP. Data are pooled from two independent experiments with 5 - 8 mice per genotype. (F) Frequency of NKT cells as defined in (C) in the liver of *Zbtb16*$^{+/tdTom}$ and *Zbtb16*$^{tdTom/tdTom}$ littermates and C57Bl/6 controls. Data pooled from four independent experiments with 12 - 15 mice per genotype. (G) Frequency of the indicated splenic lymphocytes in *Zbtb16*$^{+/tdTom}$ and *Zbtb16*$^{tdTom/tdTom}$ littermates and C57Bl/6 controls (naïve CD4+ = $CD45^{+}CD3^{+}CD4^{+}CD62L^{hi}CD44^{lo}$; activated CD4+ = $CD45^{+}CD3^{+}CD4^{+}CD62L^{lo}CD44^{hi}$; CD25+CD4+ = $CD45^{+}CD3^{+}CD4^{+}CD25^{+}$; CD8+ = $CD45^{+}CD3^{+}CD8^{+}$, $NK1.1^{+}$ = $CD45^{+}CD3^{-}CD19^{-}NK1.1^{+}$). (H) Gating strategy and Zbtb16-Tom expression status of LTi cells from *Zbtb16*$^{+/tdTom}$ embryonic liver (E15.5), as compared to WT controls. Data representative of n = 9. (D-G) Data represent mean ± SEM; not significant (ns); one-way ANOVA with *Dunnett's* post-hoc test.

Figure S6. Gating and flow cytometry analysis of progenitor-derived ILC progeny. Related to Figure 6

(A) Expression of T-bet in NK and ILC1 progeny arising from co-culture of the indicated bone marrow progenitor populations from 5x polychomILC mice with OP9 stromal cells and IL-2, IL-15, and IL-18 for 48 hrs. Data from one experiment. (B) Flow cytometric analysis of Bcl11b, Eomes, perforin, and IFN-γ expression in LiveCD45.2^{+} spleen cells stimulated with IL-2, IL-15, and IL-18, 6 weeks after transfer of IVb or IVc (CD45.2^{+}) cells into *Rag2*$^{-/-}$*Il2rgc*$^{-/-}$ recipients (CD45.1^{+}). Concatenated samples from 4 – 5 mice in 2 independent experiments

Figure S7. Extrapolation of pseudotime analysis to generate a revised model for ILC development in bone marrow. Related to Figure 7.

(A) Individual overlays of populations I, II, III, IVa, IVb and IVc onto the pseudotime trajectory shown in main Figure 7E. Cells for which no index sequencing data were available are labelled 'undetermined'. (B) Revised model for ILC development in mouse bone marrow.

Figure S1

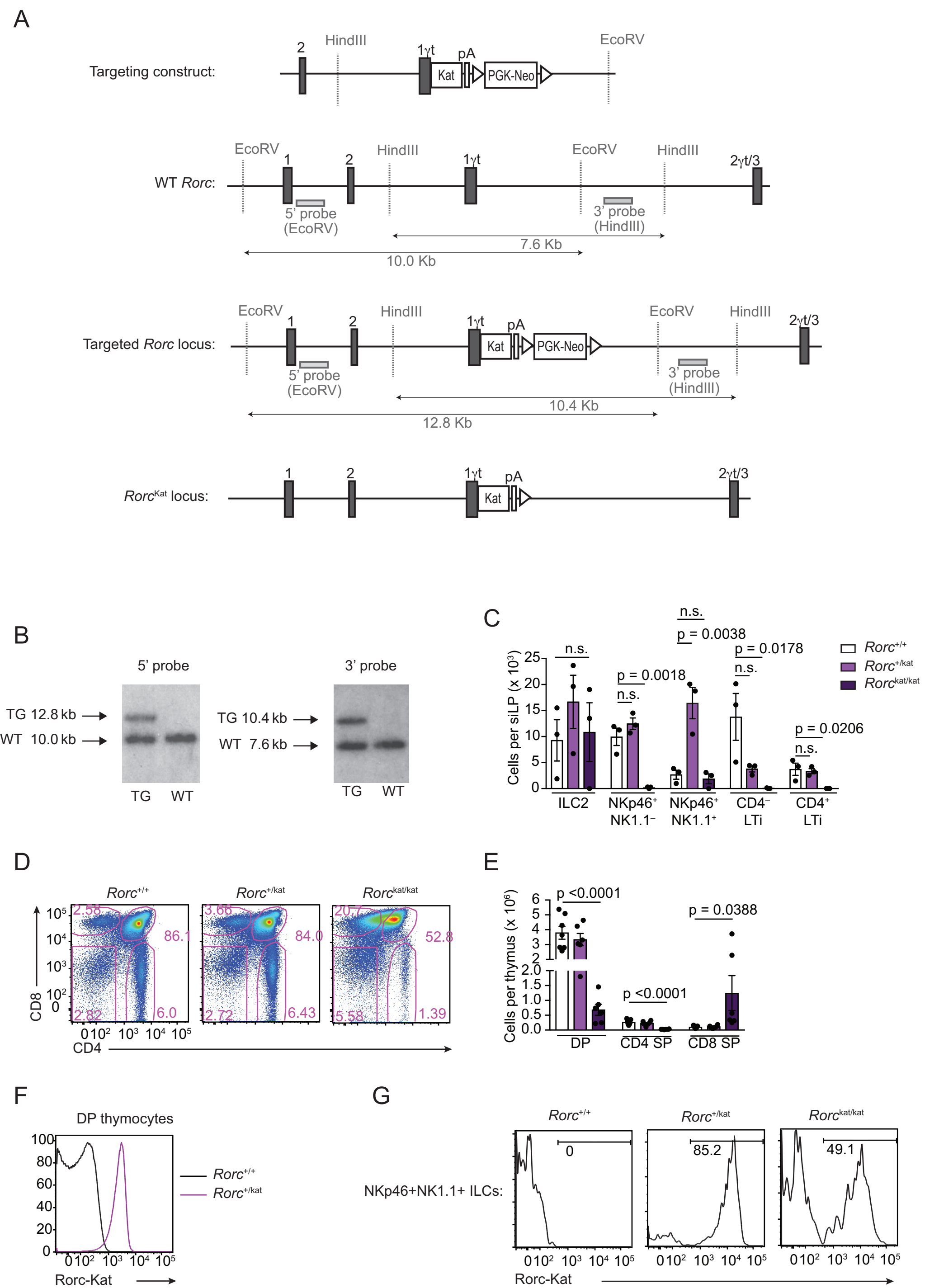
A
Targeting construct:
2
HindIII
1γt
pA
Kat
PGK-Neo
EcoRV
WT Rorc:
EcoRV
1
2
HindIII
1γt
EcoRV
HindIII
2γt/3
5' probe (EcoRV)
3' probe (HindIII)
7.6 Kb
10.0 Kb
Targeted Rorc locus:
10.4 Kb
12.8 Kb
Rorc^Kat locus:
B
5' probe
3' probe
TG 12.8 kb
WT 10.0 kb
TG 10.4 kb
WT 7.6 kb
TG
WT
C
Cells per siLP (x 10^3)
n.s.
p = 0.0018
p = 0.0038
p = 0.0178
p = 0.0206
Rorc+/+
Rorc+/kat
Rorckat/kat
ILC2
NKp46+ NK1.1–
NKp46+ NK1.1+
CD4– LTi
CD4+ LTi
D
Rorc+/+
Rorc+/kat
Rorckat/kat
2.58
86.1
2.82
6.0
3.66
84.0
2.72
6.43
20.7
52.8
5.58
1.39
CD8
CD4
E
Cells per thymus (x 10^6)
p <0.0001
p = 0.0388
DP
CD4 SP
CD8 SP
F
DP thymocytes
Rorc+/+
Rorc+/kat
Rorc-Kat
G
NKp46+NK1.1+ ILCs:
Rorc+/+
Rorc+/kat
Rorckat/kat
0
85.2
49.1
Rorc-Kat

Figure S2

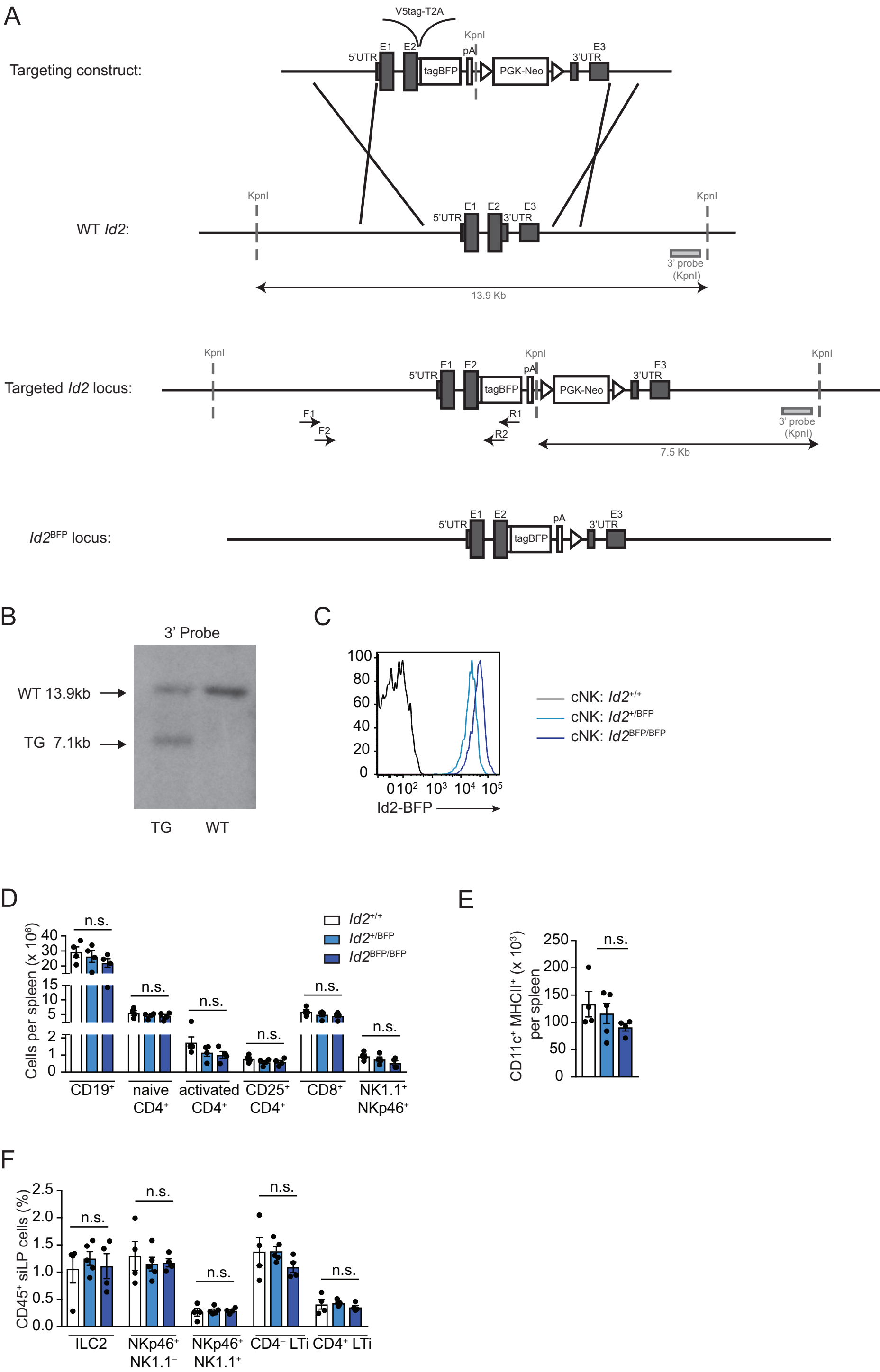

A
Targeting construct:
V5tag-T2A
5'UTR
E1
E2
tagBFP
KpnI
pA
PGK-Neo
E3
3'UTR
WT Id2:
KpnI
3' probe (KpnI)
13.9 Kb
Targeted Id2 locus:
F1
F2
R1
R2
7.5 Kb
Id2BFP locus:
B
3' Probe
WT 13.9kb
TG 7.1kb
TG
WT
C
cNK: Id2+/+
cNK: Id2+/BFP
cNK: Id2BFP/BFP
Id2-BFP
D
Cells per spleen (x 10^6)
n.s.
Id2+/+
Id2+/BFP
Id2BFP/BFP
CD19+
naive CD4+
activated CD4+
CD25+ CD4+
CD8+
NK1.1+ NKp46+
E
CD11c+ MHCII+ (x 10^3) per spleen
F
CD45+ siLP cells (%)
ILC2
NKp46+ NK1.1−
NKp46+ NK1.1+
CD4− LTi
CD4+ LTi

Figure S3

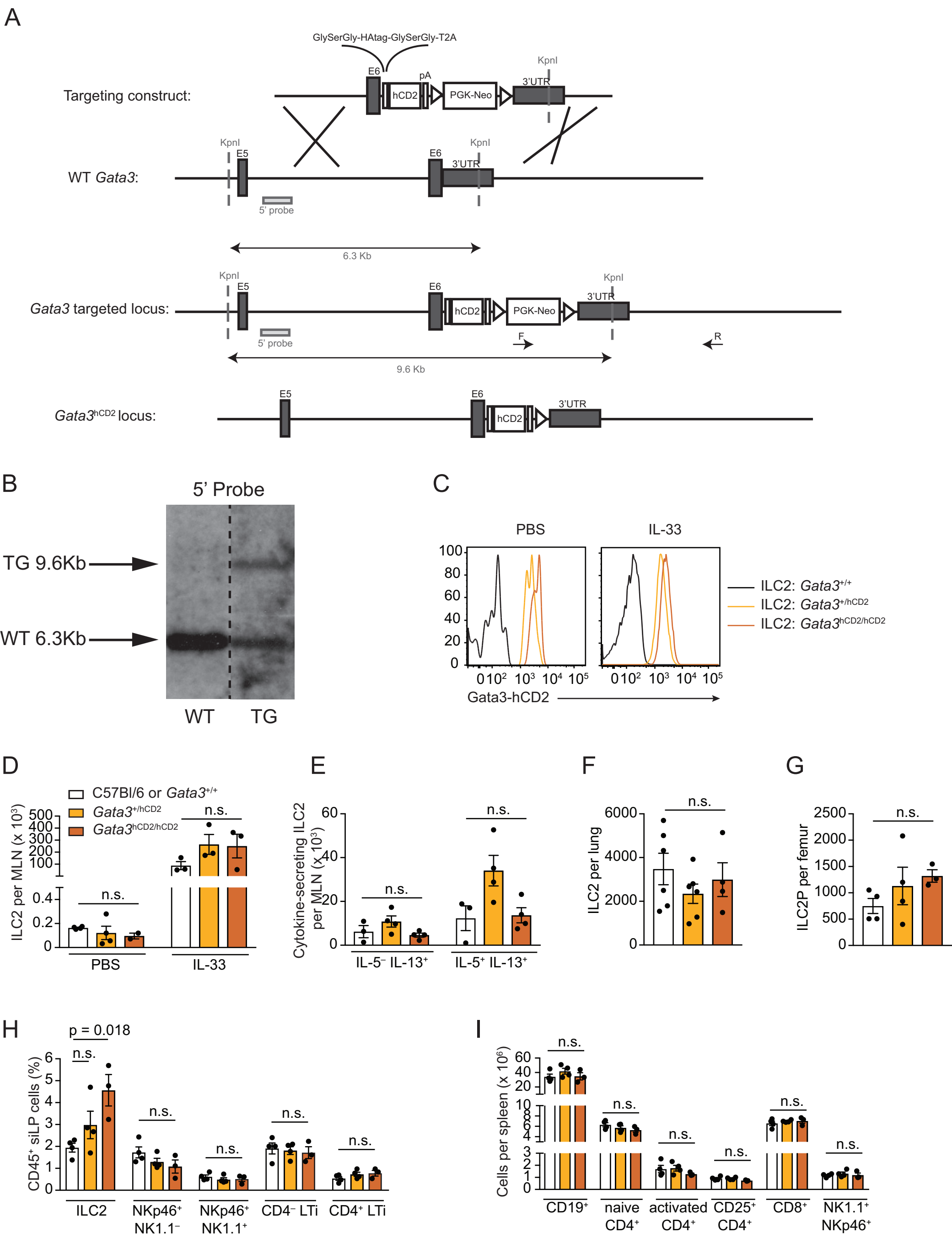
A
GlySerGly-HAtag-GlySerGly-T2A
Targeting construct:
E6
pA
hCD2
PGK-Neo
KpnI
3'UTR
WT Gata3:
KpnI
E5
E6
3'UTR
5' probe
6.3 Kb
Gata3 targeted locus:
9.6 Kb
F
R
Gata3hCD2 locus:
B
5' Probe
TG 9.6Kb
WT 6.3Kb
WT
TG
C
PBS
IL-33
Gata3-hCD2
ILC2: Gata3+/+
ILC2: Gata3+/hCD2
ILC2: Gata3hCD2/hCD2
D
C57Bl/6 or Gata3+/+
Gata3+/hCD2
Gata3hCD2/hCD2
ILC2 per MLN (x 10³)
n.s.
PBS
IL-33
E
Cytokine-secreting ILC2 per MLN (x 10³)
IL-5⁻ IL-13⁺
IL-5⁺ IL-13⁺
F
ILC2 per lung
G
ILC2P per femur
H
p = 0.018
CD45⁺ siLP cells (%)
ILC2
NKp46⁺ NK1.1⁻
NKp46⁺ NK1.1⁺
CD4⁻ LTi
CD4⁺ LTi
I
Cells per spleen (x 10⁶)
CD19⁺
naive CD4⁺
activated CD4⁺
CD25⁺ CD4⁺
CD8⁺
NK1.1⁺ NKp46⁺

Figure S4

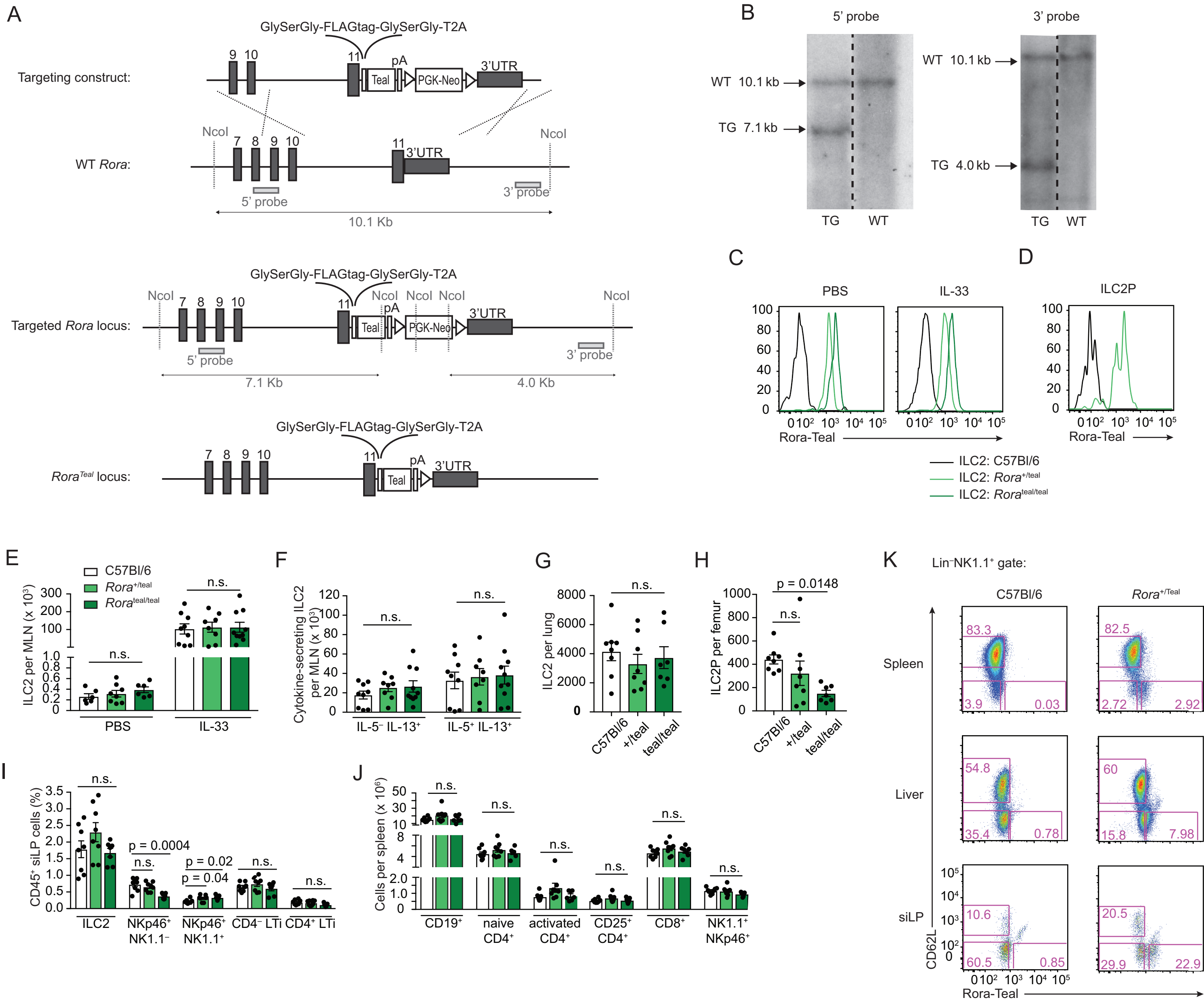

A
GlySerGly-FLAGtag-GlySerGly-T2A
Targeting construct:
9 10 11 pA Teal PGK-Neo 3'UTR
WT Rora:
NcoI 7 8 9 10 11 3'UTR NcoI
5' probe
3' probe
10.1 Kb
Targeted Rora locus:
NcoI 7 8 9 10 11 NcoI pA NcoI NcoI Teal PGK-Neo 3'UTR NcoI
5' probe
3' probe
7.1 Kb
4.0 Kb
Rora^Teal locus:
7 8 9 10 11 pA Teal 3'UTR
B
5' probe
3' probe
WT 10.1 kb
TG 7.1 kb
WT 10.1 kb
TG 4.0 kb
TG WT
TG WT
C
PBS
IL-33
Rora-Teal
D
ILC2P
Rora-Teal
ILC2: C57Bl/6
ILC2: Rora+/teal
ILC2: Rorateal/teal
E
C57Bl/6
Rora+/teal
Rorateal/teal
ILC2 per MLN (x 10³)
n.s.
PBS
IL-33
F
Cytokine-secreting ILC2 per MLN (x 10³)
n.s.
IL-5⁻ IL-13⁺
IL-5⁺ IL-13⁺
G
ILC2 per lung
n.s.
C57Bl/6
+/teal
teal/teal
H
ILC2P per femur
p = 0.0148
n.s.
C57Bl/6
+/teal
teal/teal
I
CD45⁺ siLP cells (%)
n.s.
p = 0.0004
p = 0.02
p = 0.04
ILC2
NKp46⁺ NK1.1⁻
NKp46⁺ NK1.1⁺
CD4⁻ LTi
CD4⁺ LTi
J
Cells per spleen (x 10⁶)
n.s.
CD19⁺
naive CD4⁺
activated CD4⁺
CD25⁺ CD4⁺
CD8⁺
NK1.1⁺ NKp46⁺
K
Lin⁻NK1.1⁺ gate:
C57Bl/6
Rora+/Teal
Spleen
83.3
3.9
0.03
82.5
2.72
2.92
Liver
54.8
35.4
0.78
60
15.8
7.98
siLP
10.6
60.5
0.85
20.5
29.9
22.9
CD62L
Rora-Teal

Figure S5

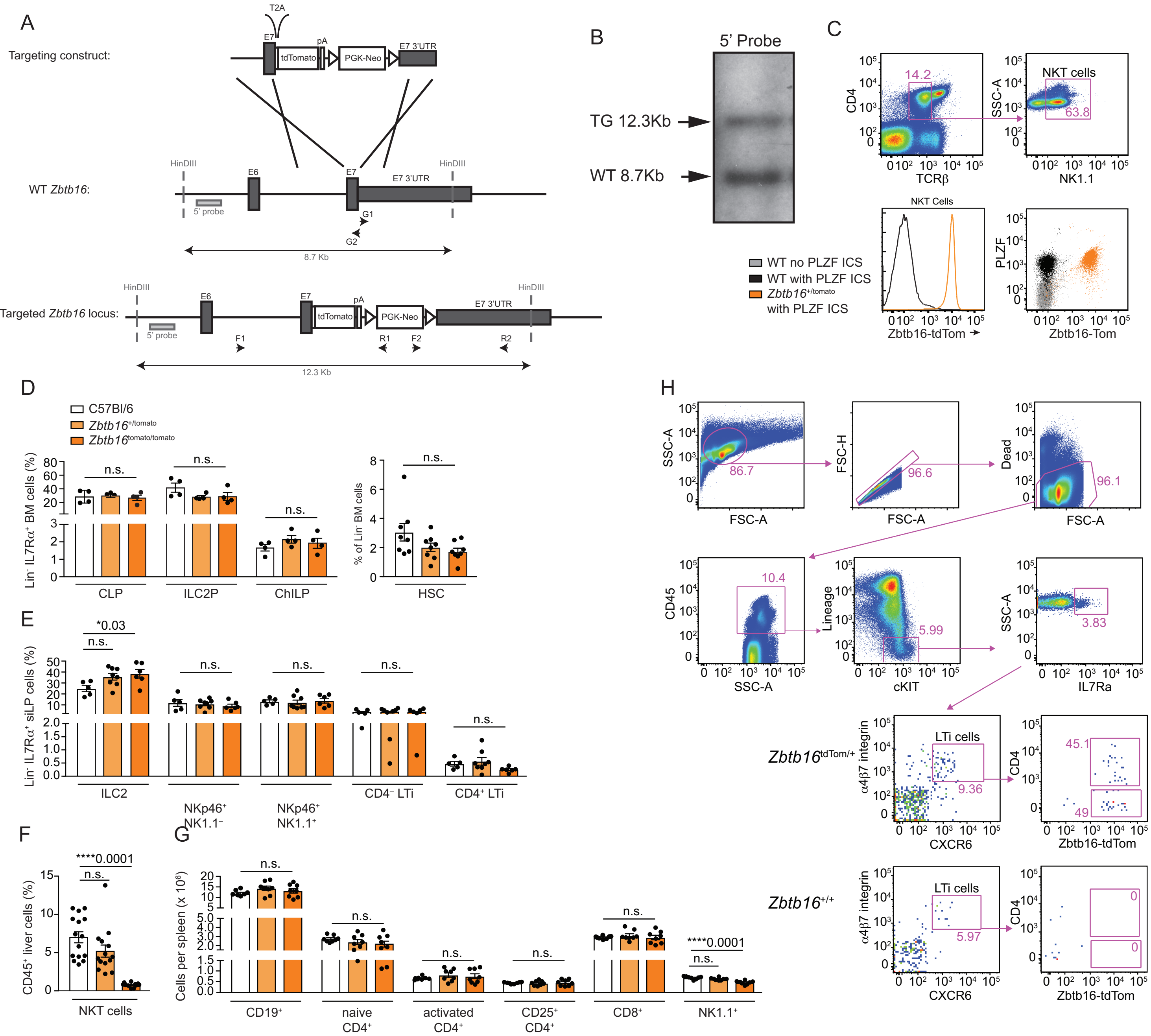
A
Targeting construct:
T2A
E7
tdTomato
pA
PGK-Neo
E7 3'UTR
WT Zbtb16:
HinDIII
E6
E7
E7 3'UTR
HinDIII
5' probe
G1
G2
8.7 Kb
Targeted Zbtb16 locus:
HinDIII
E6
E7
tdTomato
pA
PGK-Neo
E7 3'UTR
HinDIII
5' probe
F1
R1
F2
R2
12.3 Kb
B
5' Probe
TG 12.3Kb
WT 8.7Kb
C
14.2
CD4
TCRβ
NKT cells
63.8
SSC-A
NK1.1
NKT Cells
WT no PLZF ICS
WT with PLZF ICS
Zbtb16+/tomato with PLZF ICS
Zbtb16-tdTom
PLZF
Zbtb16-Tom
D
C57Bl/6
Zbtb16+/tomato
Zbtb16tomato/tomato
Lin- IL7Rα+ BM cells (%)
n.s.
CLP
ILC2P
ChILP
% of Lin- BM cells
HSC
E
*0.03
n.s.
Lin- IL7Rα+ siLP cells (%)
ILC2
NKp46+ NK1.1−
NKp46+ NK1.1+
CD4− LTi
CD4+ LTi
F
****0.0001
n.s.
CD45+ liver cells (%)
NKT cells
G
Cells per spleen (x 10^6)
CD19+
naive CD4+
activated CD4+
CD25+ CD4+
CD8+
NK1.1+
****0.0001
H
86.7
SSC-A
FSC-A
96.6
FSC-H
FSC-A
96.1
Dead
FSC-A
10.4
CD45
SSC-A
5.99
Lineage
cKIT
3.83
SSC-A
IL7Ra
Zbtb16tdTom/+
LTi cells
9.36
α4β7 integrin
CXCR6
45.1
49
CD4
Zbtb16-tdTom
Zbtb16+/+
LTi cells
5.97
α4β7 integrin
CXCR6
0
0
CD4
Zbtb16-tdTom

Figure S6

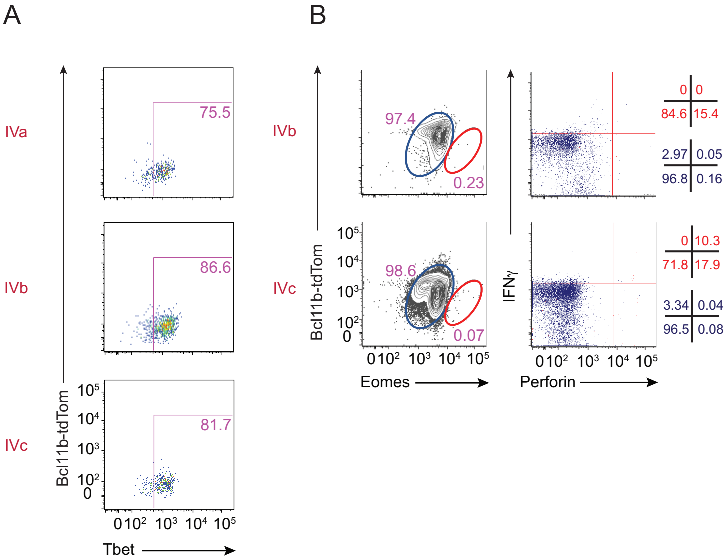

A
B
IVa
IVb
IVc
75.5
86.6
81.7
Bcl11b-tdTom
Tbet
97.4
0.23
98.6
0.07
Eomes
IFNγ
Perforin
0 0
84.6 15.4
2.97 0.05
96.8 0.16
0 10.3
71.8 17.9
3.34 0.04
96.5 0.08

Figure S7

A

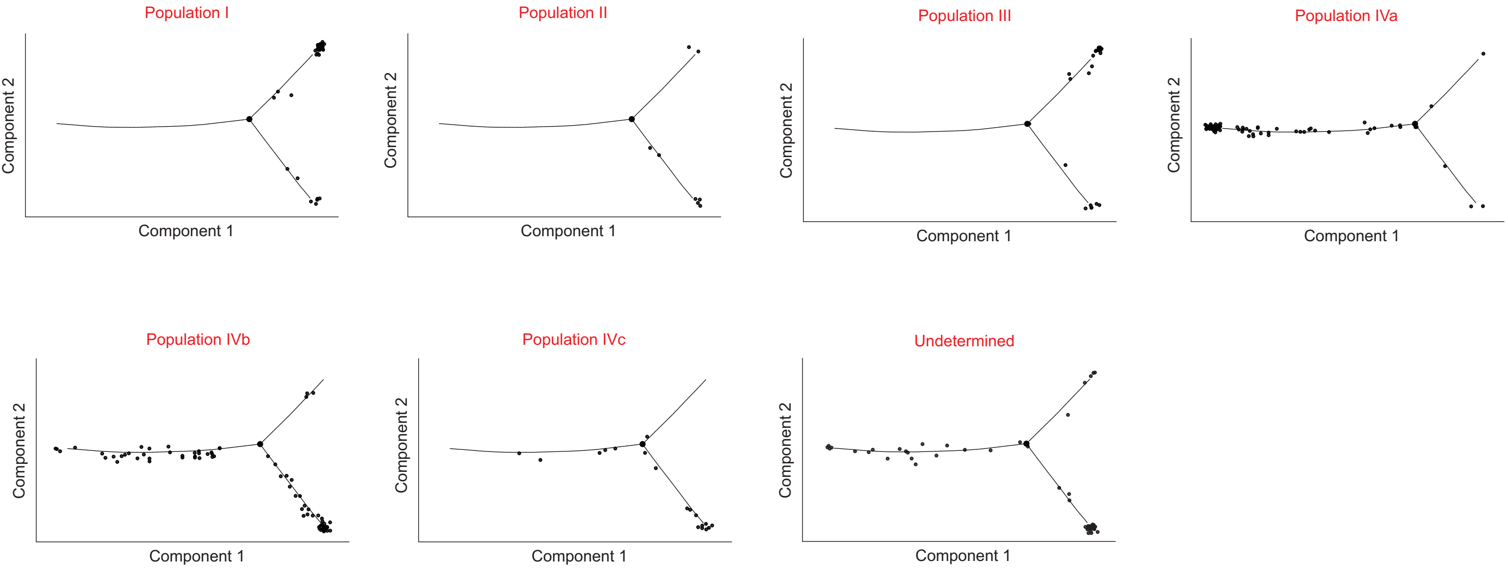

Population I
Component 2
Component 1
Population II
Component 2
Component 1
Population III
Component 2
Component 1
Population IVa
Component 2
Component 1
Population IVb
Component 2
Component 1
Population IVc
Component 2
Component 1
Undetermined
Component 2
Component 1

B

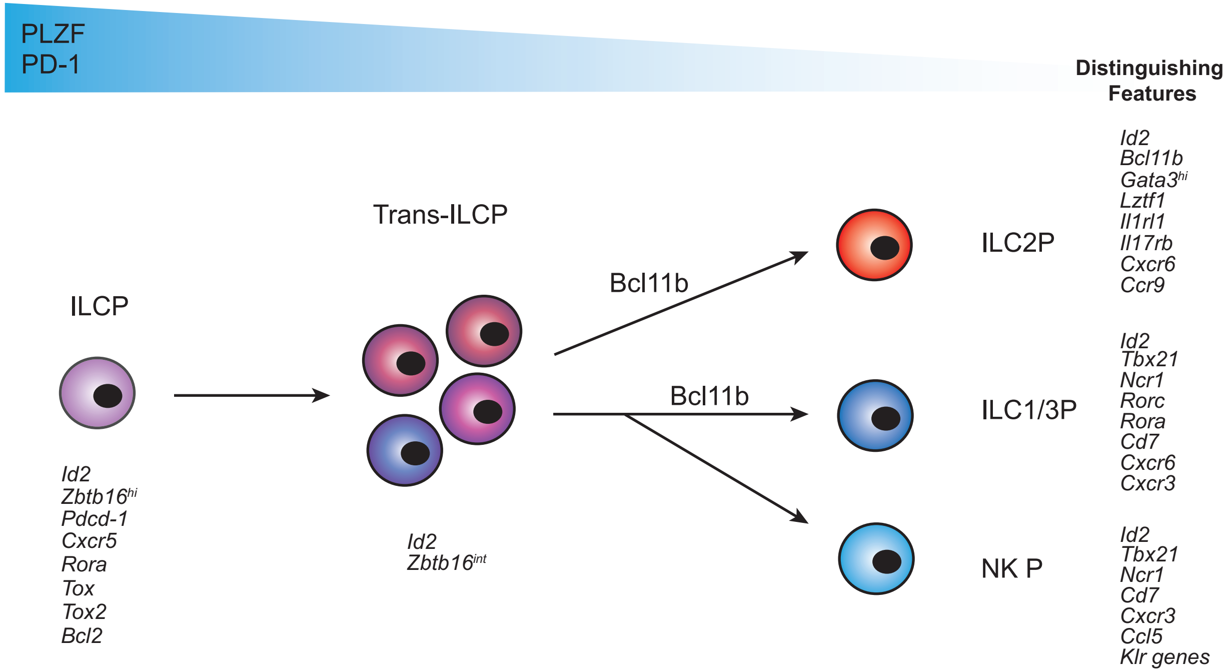

PLZF
PD-1
Distinguishing Features
ILCP
Id2
Zbtb16hi
Pdcd-1
Cxcr5
Rora
Tox
Tox2
Bcl2
Trans-ILCP
Id2
Zbtb16int
Bcl11b
Bcl11b
ILC2P
Id2
Bcl11b
Gata3hi
Lztf1
Il1rl1
Il17rb
Cxcr6
Ccr9
ILC1/3P
Id2
Tbx21
Ncr1
Rorc
Rora
Cd7
Cxcr6
Cxcr3
NK P
Id2
Tbx21
Ncr1
Cd7
Cxcr3
Ccl5
Klr genes

Table S1. ILC progenitor nomenclature. Related to Figures 3 and 4.

Progenitor population	Progenitor phenotype	In vivo progeny	In vitro progeny
I	$\mathrm{Lin^{-}IL\text{-}7R\alpha^{+}Id2^{+}Bcl11b^{+}CD25^{+}Gata3^{hi}\alpha4\beta7^{int}}$	ILC2	ILC2
II	$\mathrm{Lin^{-}IL\text{-}7R\alpha^{+}Id2^{+}Bcl11b^{+}CD25^{-}Gata3^{lo}\alpha4\beta7^{lo}}$	ILC2	ILC2
III-lo	$\mathrm{Lin^{-}IL\text{-}7R\alpha^{+}Id2^{+}Bcl11b^{+}CD25^{-}Gata3^{lo}\alpha4\beta7^{lo}}$	NK + ILC2 (few ILC1/ILC2)	ILC1,ILC2,ILC3
III-hi	$\mathrm{Lin^{-}IL\text{-}7R\alpha^{+}Id2^{+}Bcl11b^{+}CD25^{-}Gata3^{hi}\alpha4\beta7^{int}}$	ILC2	ILC2
III-lo-kat^{+}	$\mathrm{Lin^{-}IL\text{-}7R\alpha^{+}Id2^{+}Bcl11b^{+}CD25^{-}Gata3^{lo}\alpha4\beta7^{int}Rorc^{+}}$	ILC2 + ILC3	ILC3
IV-lo	$\mathrm{Lin^{-}IL\text{-}7R\alpha^{+}Id2^{+}Bcl11b^{-}CD25^{-}Gata3^{lo}\alpha4\beta7^{mix}}$	NK,ILC1,ILC2,ILC3	Not done
IV-hi	$\mathrm{Lin^{-}IL\text{-}7R\alpha^{+}Id2^{+}Bcl11b^{-}CD25^{-}Gata3^{hi}\alpha4\beta7^{mix}}$	ILC2 + ILC3	Not done
IVa	$\mathrm{Lin^{-}IL\text{-}7R\alpha^{+}Id2^{+}Bcl11b^{-}CD25^{-}Gata3^{hi}\alpha4\beta7^{hi}}$	NK,ILC1,ILC2,ILC3	ILC1 + ILC2
IVb	$\mathrm{Lin^{-}IL\text{-}7R\alpha^{+}Id2^{+}Bcl11b^{-}CD25^{-}Gata3^{hi}\alpha4\beta7^{lo}Rorc^{-}}$	NK,ILC1,ILC2,ILC3	ILC1 + ILC2
IVc	$\mathrm{Lin^{-}IL\text{-}7R\alpha^{+}Id2^{+}Bcl11b^{-}CD25^{-}Gata3^{hi}\alpha4\beta7^{lo}Rorc^{+}}$	NK,ILC1,ILC2,ILC3	ILC1,ILC2,ILC3
		**PLZF expression**	**Chemokine receptors**
EILP (a)	$\mathrm{Lin^{-}IL\text{-}7R\alpha^{lo}Thy1^{-}\alpha4\beta7^{+}Kit^{+}CD122^{lo}TCF7^{+}}$	$\mathrm{PLZF^{int}}$	
αLP (b)	$\mathrm{Lin^{-}IL\text{-}7R\alpha^{+}Id2^{+}CD25^{-}\alpha4\beta7^{+}Flt3^{-}Kit^{int}Sca1^{int}}$	$\mathrm{PLZF^{+/-}}$	$\mathrm{CXCR6^{+/-}}$
CHILP (c)	$\mathrm{Lin^{-}IL\text{-}7R\alpha^{+}Id2^{+}CD25^{-}\alpha4\beta7^{hi}Flt3^{-}}$	$\mathrm{PLZF^{+/-}}$	
ILCP (a)	$\mathrm{Lin^{-}IL\text{-}7R\alpha^{+}Thy1^{+}\alpha4\beta7^{hi}Flt3^{-}Kit^{+}CD122^{lo}TCF7^{+}}$	$\mathrm{PLZF^{hi}}$	$\mathrm{CXCR5^{+}}$, $\mathrm{CXCR6^{+}}$

(a) (Harly et al., 2018) (b) (Seillet et al., 2016) (c) (Klose et al., 2014)
